# Cargo Analysis and MRI‐Based Therapeutic Assessment of Iron Oxide Labelled Extracellular Vesicles of Hypoxia Human Stem Cells in Ischemic Stroke

**DOI:** 10.1002/jex2.70063

**Published:** 2025-07-17

**Authors:** Shannon Helsper, Li Sun, Richard Jeske, Chang Liu, Jacob Athey, Xuegang Yuan, Samuel C. Grant, Yan Li

**Affiliations:** ^1^ Chemical & Biomedical Engineering, FAMU‐FSU College of Engineering Florida State University Tallahassee Florida USA; ^2^ National High Magnetic Field Laboratory Florida State University Tallahassee Florida USA; ^3^ Department of Biomedical Sciences Florida State University College of Medicine Tallahassee Florida USA

**Keywords:** extracellular vesicles, human mesenchymal stem cells, hypoxia, ischemia, magnetic resonance imaging

## Abstract

Human mesenchymal stem cells (hMSCs) have been under investigation in preclinical and clinical settings for treating neurological disorders in recent years. Predominantly due to paracrine effects *in vivo*, hMSC‐secreted extracellular vesicles (EVs) are at the forefront of these investigations. In this study, the therapeutic efficacy of hypoxia hMSCs and the secreted EVs labelled with iron oxides was evaluated in a preclinical model of ischemic stroke. Transcriptome and proteomics analysis of hMSCs under hypoxia indicated alterations in metabolic pathways and EV biogenesis. Hypoxia preconditioning increased EV yield by 57% with similar EV size and exosomal marker expression. EV cargo analysis using proteomics and microRNA‐sequencing revealed that hypoxia preconditioning upregulated expression of metabolic proteins related to hypoxia‐inducible factor signalling, neurogenesis and EV biogenesis. Magnetic resonance imaging following *in vivo* administration of iron oxide‐labelled hMSCs and EVs provided assessment of biodistribution and therapeutic efficacy. The results indicated differential recovery in sodium levels in rats following hMSC and EV administration compared to the vehicle‐only group, supported by lactate levels and functional assessment. hMSC‐EVs localized to the ischemic lesion and evoked a therapeutic response after a single bolus injection. This study has significance in developing human stem cell‐free therapeutics for treating ischemic stroke.

**Abbreviations**
CSI: chemical shift imaging
DEG: differentially expressed gene
DEP: differentially expressed protein
ECM: extracellular matrix
ESCRT: endosomal sorting complex responsible for transport
EV: extracellular vesicle
FBS: foetal bovine serum
FSE: fast spin echo
FSU: Florida State University
GO: gene ontology
HIF: hypoxia‐inducible factor
hMSC: human mesenchymal stem cells
KEGG: Kyoto Encyclopaedia of Genes and Genomes
MACO: middle cerebral artery occlusion
MPIO: micron‐size particles of iron oxide
MRI: magnetic resonance imaging
MRS: magnetic resonance spectroscopy
MVB: multivesicular bodies
NF‐kB: nuclear factor kappa‐light‐chain‐enhancer of activated B
NTA: nanoparticle tracking analysis
PBS: phosphate buffered saline
PCA: principal component analysis
PEG: polyethylene glycol
TBS‐T: tris‐buffered saline with 0.1% Tween 20 (v/v)
TEM: transmission electron microscopy
TNF: tumour necrosis factor
USPIO: ultrasmall iron oxide nanoparticles

## 1 Introduction

Clinical studies have investigated bone marrow derived human mesenchymal stem cells (hMSCs) to assess their therapeutic efficacy for a wide range of pathologies including osteoarthritis, diabetes, acute respiratory distress syndrome, spinal cord injury, multiple sclerosis and stroke (https://www.clinicaltrials.gov). hMSCs have been shown to play regulatory roles in cell‐to‐cell communications and substantially affect the recruitment of endogenous neural progenitor cells (Arvidsson et al. 2002). The underlying mechanism when hMSCs perform these functions; however, is not yet understood fully. However, the release of small extracellular vesicles (EVs) (50–200 nm, e.g., exosomes that are generated in the endosomal compartment of the cells) has been identified as key to these processes.

EVs are membrane‐bound vesicles released from parent cells including hMSCs due to multivesicular bodies (MVB)‐related proteins associated with the endosomal sorting complex responsible for transport (ESCRT) (Hong et al. 2019; Raposo and Stoorvogel 2013). Beyond biogenesis pathway, several factors distinguish small EVs, in particular exosomes, from other vesicular bodies, including size (50–200 nm in diameter) and specific protein markers (e.g., HSC70, CD81, CD63, Alix and TSG101) (Otero‐Ortega et al. 2019). The innate immunomodulatory and regulatory functions of hMSC‐derived EVs aid in many pathologies including cardiovascular, liver and kidney diseases (Valadi et al. 2007; Vlassov et al. 2012; Xin et al. 2013). In rodent models of ischemia, systemic administration of EVs has promoted functional recovery, and neuroregeneration (Xin et al. 2013; Doeppner et al. 2015). Their protein cargo is suspected to impact synaptic transmission, neuronal differentiation, angiogenesis and neuronal projections (Otero‐Ortega et al. 2019). In addition, functional exosomal miRNA transferred by EVs between hMSCs and neural cells promote neurite remodelling, functional recovery (Xin et al. 2012) and prevent post‐ischemic immunosuppression (Doeppner et al. 2015). Similar to the direct homing capacity of hMSCs to lesions, EVs also tend to target regions of neurodegeneration (Perets et al. 2019). Longitudinal evaluations of tissue recovery resulting from the delivery of EVs compared directly to their cellular counterparts remain essential to improve understanding of the therapeutic mechanism and potentials underlying hMSC implantation.

The goal of this study was to establish whether the impact of hMSC therapy applied to cerebral ischemia is through secreted EVs rather than direct cell–cell interactions. EVs can potentially be utilized to deliver higher concentrations of therapeutic content such as miRNAs (Xin et al. 2012; Zhang et al. 2017) and proteins related to regenerative processes, in a more controlled manner without inducing immune response than the parent cells (Otero‐Ortega et al. 2019). EVs are typically considered safer than the parent cells since they are not capable of uncontrolled proliferation or differentiation. In addition, previous studies have demonstrated enhanced therapeutic efficacy resulting from various hMSC culture preconditioning, including hypoxia and three‐dimensional culture (Helsper et al. 2022, 2023; Rosenberg et al. 2016). In line with enhanced stem cell properties, preconditioning of hMSCs also alters EV yield, content and homing abilities, potentially expanding their therapeutic utility (Yuan et al. 2019; Yuan et al. 2022). Thus, this study utilized hypoxia‐preconditioning of hMSCs and isolated the secreted EVs to evaluate the potential regenerative effects on ischemia injury.

Several isolation techniques to harvest EVs have been investigated over the years including ultracentrifugation (Linares et al. 2015), size exclusion chromatography (Otero‐Ortega et al. 2019) and precipitation by polyethylene glycol (PEG) (Rider et al. 2016). The ExtraPEG approach is particularly advantageous over commercial kits due to its enrichment method, allowing large media volumes to be processed with a single ultra‐centrifugation step (Rider et al. 2016). Beyond EV harvesting, another significant challenge to overcome in studying their use as a novel therapeutic has been visualizing and tracking EVs *in vivo*, providing insight into initial biodistribution upon delivery. Various techniques, including bioluminescence (Lai et al. 2014; Imai et al. 2015), fluorescence (Grange et al. 2014; Antes et al. 2018) and magnetic resonance imaging (MRI) (Han et al. 2021; Dabrowska et al. 2018; Zhi et al. 2020; Busato et al. 2017; Helsper et al. 2024), have been used for *in vivo* EV tracking. Specifically, MRI is advantageous over alternative imaging modalities for therapy tracking due to its ability to evaluate tissue recovery in response to therapeutic administration. Nanoscale iron oxide particles have been used to label hMSCs for MRI visualization, albeit with not all particle sizes feasible for labelling EVs (Rosenberg et al. 2013, 2014). Sonication has been used successfully to load EVs with small‐molecule drugs and proteins without damaging EV integrity (Zeng et al. 2023; Colja et al. 2023). Recently, an adaptation of the ExtraPEG enrichment purification method was developed in our lab to optimize EV labelling with ultrasmall iron oxide nanoparticles (USPIO) (Helsper et al. 2024), an effective MRI contrast. However, the influence of USPIO labelling on EV cargo and the therapeutic effects *in vivo* have not been well investigated.

Following hMSC or EV labelling, *in vivo* ^23^Na and ^1^H MRI and spectroscopy (MRS) methods were used in this study to evaluate the efficacy of novel hMSC‐EV therapies in a preclinical model of cerebral stroke. During an ischemic event, disrupted blood flow dysregulates Na^+^/K^+^‐ATPase (Madelin et al. 2014), leading to osmotic swelling and ionic homeostatic disruption that culminates in neuronal death. Sodium, as a quantifiable metric of ischemic lesion and tissue recovery, is linked to this acute disruption. High resolution ^23^Na MRI permits assessment of bulk sodium content in the ischemic core and penumbra while providing excellent anatomical referencing, with and without conventional ^1^H MRI (Abad et al. 2018). Selective and localized *in vivo* ^1^H MRS compliment sodium MRI by tracking changes in energetics, osmoregulation and neurotransmitters (Helsper et al. 2023; Shemesh et al. 2013; Shemesh et al. 2014; Rosenberg et al. 2017). MRI/S at ultra‐high field enable tracking of hMSCs and the secreted EVs to reveal the correlation of functional biochemical changes with neuromotor outcomes and proteomic data. To determine the contribution of hMSC‐EVs to the tissue recovery after ischemia in stroke, hMSCs grown under hypoxia and their derived EVs were administered to an ischemic stroke rat model after USPIO labelling and assessed using MRI metrics in this study. In particular, the correlation between EV protein and miRNA cargo and the therapeutic benefits was revealed. This study has the significance in furthering the development of EV‐based therapies for treating ischemic stroke.

## 2 Materials and Methods

### 2.1 Human Stem Cell Culture

hMSCs isolated from bone marrow (Tulane Centre for Gene Therapy) were cultured in complete culture media containing α‐MEM with 10% foetal bovine serum (FBS, Atlanta Biologicals, Lawrenceville, GA) and 1% Penicillin/Streptomycin (Life Technologies, Carlsbad, CA) with media change every 3 days. Three donors were used for hMSC culture and EV collection (**Table** **S1**). At passage 4, media were changed to EV‐depleted media and either maintained under normoxic conditions or placed into a hypoxia chamber under 2% O_2_, 5% CO_2_ (BioSpherix Ltd., Parish, NY) for 1 week. Media‐containing EV were harvested on days 4, 5 and 6. Before transplantation, hMSCs were incubated with micron‐size particles of iron oxide (MPIO, 7.47‐µg Fe/mL, 0.86 µm with encapsulated carboxyl modified polystyrene (P(S/V‐COOH)), part number ME03F/9772, Bangs Laboratories Inc., Fishers, IN) for 12 h according to previously published methods (Rosenberg et al. 2016). Subsequent rinsing and re‐suspension of the hMSC in phosphate buffered saline (PBS) was performed just prior to injection.

### 2.2 hMSC‐EV Isolation

EVs were isolated from culture medium following a sequential spin series (500 g for 5 min, 2000 g for 10 min, 10,000 g for 30 min at 4°C) to remove cell debris, apoptotic body and large vesicles. EVs were enriched using an ExtraPEG method as previously outlined (Rider et al. 2016) by adding 16% PEG‐6000 (81260 Sigma Aldrich, St. Louis, MO) to the final supernatant followed by storing at 4°C for 24 h. Another low‐speed centrifugation was completed to obtain the pelleted EVs (3000 g for 60 min). The pellet was suspended in 1 mL PBS and prepared for ultracentrifugation at 127,000 g for 70 min at 4°C, using the SW‐28 swing‐bucket rotor in an Optima XL‐100K ultracentrifuge (k factor‐15, Beckman Coulter, Inc., Pasadena, CA). Afterward, the EV pellet was suspended in 100 µL PBS, and a benchtop shaker at 1500 rpm for 5 min was used to disrupt the pellet. For *in vitro* analysis, EV samples were stored at −80°C until used. For *in vivo* injection, EV samples were stored a 4°C and used within 24 h. EVs from three different hMSC donors were used.

### 2.3 Nanoparticle Tracking Analysis (NTA)

NTA was used to quantify EV size distribution and concentration. All samples were repeated in triplicate using Nanosight (NTA 3.4 Build 3.4.003, Salisbury, UK). First, EV samples (10^8^–10^9^ count) were diluted by adding 1 µL of EV solution into 1 mL Millipore water. The camera sCMOS was set at level 13, and a detection threshold set to four. Merged data from the triplicates were calculated for mean, mode, size thresholds D10, D50 and D90 (10%, 50% and 90%, respectively), standard deviation and particle concentration (particles per mL).

### 2.4 Transmission Electron Microscopy (TEM)

Electron microscopy imaging was performed to confirm EV morphology. Briefly, intact EVs (5 µL) were dropped onto parafilm. A carbon coated 400 Hex Mesh Copper grid (Electron Microscopy Sciences, EMS, Hatfield, PA) was positioned using forceps with coating side down on top of each drop for 1 h. Grids were washed with sterile filtered PBS three times, and then the EV samples were fixed for 10 min in 2% paraformaldehyde (EM grade, EMS). After washing, the grids were transferred on top of a 20‐µL drop of 2.5% glutaraldehyde (EM Grade, EMS) and incubated for 10 min at room temperature. Grid samples were stained for 10 min with 2% uranyl acetate (EM grade, EMS). Then the samples were embedded for 10 min with 0.13% methyl cellulose and 0.4% uranyl acetate. The coated side of the grids were left to dry before imaging on the CM120 Biotwin electron microscope (Field Electron and Ion Company, FEI, Hillsboro, OR) at FSU Biological Science Imaging Resource.

### 2.5 Western Blot for EV Markers

EV pellets following ultracentrifugation were lysed in 2× Laemmli sample buffer (4% SDS, 100‐mM Tris‐HCl [pH 6.8], 0.4‐mg/mL bromophenol blue, 20% glycerol) for immunoblot analysis. The supernatant was collected, and a Bradford assay was carried out to determine the protein concentration. Protein lysate concentration was normalized, and 20 µg of each sample was denatured at 95°C in Laemmli Sample buffer with 2‐mercaptoethanol. Proteins were separated by 12% Bis‐Tris SDS‐PAGE gel and transferred onto a nitrocellulose membrane (Bio‐rad, Hercules, CA). The membranes were blocked for 60 min in 5% skim milk power (w/v) in Tris‐buffered saline (10‐mM Tris‐HCl [pH 7.5] and 150‐mM NaCl) with 0.1% Tween 20 (v/v) (TBS‐T). Membranes were incubated overnight in the presence of the primary antibodies (calnexin, 2433S, 1:1000; CD81, 56039S, 1:1000, Cell Signalling Technology, Danvers, MA; HSC70, 100‐401‐F49, 1:1500, Rockland Immunochemicals Inc., Limerick, PA) diluted in the blocking buffer at 4°C. Afterward, the membranes were washed four times for 10 min each with TBS‐T and then incubated with IRDye 800CW goat anti‐rabbit IgG secondary antibody (926‐32211, 1:5000, LICOR, Lincoln, NV) for 90 min at room temperature. After wash with TBS‐T, the blots were imaged using an Image Quant LAS4000 (GE Life Science, Marlboro, MA) and processed with ImageQuant TL v8.1.0.0 software.

### 2.6 mRNA‐Sequencing of hMSCs

Total RNA was extracted from hMSCs under hypoxia and normoxia (three replicates for each group) using Trizol (15596026, ThermoFisher Scientific, Waltham, MA). mRNA was isolated from the total RNA using an NEBNext Poly(A) mRNA Magnetic Isolation Module (E7490, New England Biolabs, Ipswitch, MA). cDNA libraries were generated from the isolated mRNA using an NEBNext Ultra RNA library prep kit for Illumina (E7530, New England Biolabs) and a unique six nucleotide index primer was incorporated into each sample. The library construction was done according to NEB manuals. The multiplexed sample was quantified with KAPA qPCR (KR0405, Kapa Biosystems, Hertfordshire, UK) specific for Illumina sequencing primers, and the average fragment size was determined with a Bioanalyzer high sensitivity DNA chip (5067‐4626, Agilent Technologies, Santa Clara, CA). Pooled samples were sequenced with single end, 100 base reads on an Illumina NovaSeq 6000 system (Cambridge, UK) located in the Translational Science Laboratory (TSL) at the College of Medicine, Florida State University (FSU).

The pooled data were demultiplexed into individual sample data, and adapter primer sequences were removed. Total gene counts data were analysed online with NetworkAnalyst 3.0 (Zhou et al. 2019). Genes with counts less than 10, variance percentile rank lower than 10% or unannotated were filtered, and the remainder were normalized by Log2‐counts per million reads. Differentially expressed genes (DEGs) were identified by DEseq2. Heatmaps of global DEGs and gene enriched pathways were visualized by the same online tool. The DEGs that were upregulated and downregulated between hypoxia versus normoxia groups were assessed for Gene Ontology (GO), Kyoto Encyclopaedia of Genes and Genomes (KEGG) pathway and phenotype pathway analysis by g:Profiler (version e104_eg51_p15_3922dba).

### 2.7 Proteomics Analysis for hMSCs and hMSC‐EVs Before and After Sonication

The hypoxia and normoxia hMSCs (group I and II, respectively) were extracted for proteins. In addition, the hypoxia and normoxia hMSC‐EVs (group III and IV, respectively) were isolated using ExtraPEG and then extracted for proteins along with hypoxia hMSC‐EVs after sonication (group V). Based on protein quantification results, approximately 20 µg proteins were isolated on an S‐trap micro column (K02‐micro, Protifi, Farmingdale, NY). The isolated proteins (duplicates for each group) were alkylated and digested on column based on manufacturer's instructions. All the eluted peptides were fractionated by a Pierce high pH reverse phase peptide fractionation kit (84868, ThermoFisher Scientific) into three fractions for each sample. Then all the samples were vacuumed dried and submitted to the FSU TSL for mass spectrometry. The samples were analysed on the Thermo Scientific Q Exactive HF Hybrid Quadrupole‐Orbitrap Mass Spectrometer as previously described (Nkosi et al. 2021). Briefly, resulting raw files were searched with Proteome Discoverer 2.4 (Thermo Fisher Scientific) using SequestHT, Mascot and Amanda as search engines. Scaffold 5 (Proteome Software, Portland, OR) was used to validate the protein and peptide identity. Peptide identity was accepted if the Scaffold Local FDR algorithm demonstrated a probability greater than 99.0%. Likewise, protein identity was accepted if the probability level was greater than 99.0% and contained a minimum of two recognized peptides. GO annotation was carried out by g:Profiler (Raudvere et al. 2019).

### 2.8 microRNA‐Sequencing (miRNA‐seq) Analysis for Hypoxia hMSC‐EVs

EV‐associated miRs were isolated and sequenced using the protocol below. Each sample was analysed in triplicates. EV samples were treated with RNase (ThermoFisher; AM2294) at a final concentration of 50 ng/mL, at room temperature for 30 min. RNase inhibitor (NEB; M0314) and PCR grade water were added to EV samples to make total volume of 200 µL. Total RNAs were isolated by adding 600 µL Trizol LS (ThermoFisher; 10296010) according to manufacturer's instruction. To increase the yield of small RNAs, three volumes of 100% ethanol and linear acrylamide (VWR; 97063–560) were used instead of isopropyl alcohol and incubation time was also increased to overnight at −20°C. The isolated RNAs were quantified by Qubit microRNA assay kit (ThermoFisher; Q32880). Small RNA libraries were generated with NEBNext Multiplex Small RNA Library Prep Set for Illumina (NEB; E7300). To increase yield and prevent primer/adaptor dimer, 3’ SR and all other primers was diluted to 1:10 and increase ligation time to overnight at 16°C. Similar to mRNA‐seq library preparation, HS DNA chip and KAPA library quantification kit were used before submitting to sequencing by illumina NovaSeq 6000 in Florida State University College of Medicine TSL.

Raw data for miRNA‐seq were submitted to OASIS online miRNA analysis tool to identify small RNAs on Human reference genome hg38. Differentially expressed miRNAs were analysed by both OASIS and miRNet using default settings. RNA‐seq data were analysed by NetworkAnalyst 3.0 (Zhou et al. 2019). Genes with counts less than 10, variance less than 10% and unannotated were filtered and normalized by Log2‐counts per million. DEGs were identified by DEseq2. Heatmap of global DEGs and gene enriched pathways were also visualized by the same online tool.

### 2.9 Labelling of hMSC‐EVs With Iron Oxides

EVs were labelled accordingly. In brief, USPIO purchased from Sigma Aldrich (725331‐5ML, 5 nm in average, the ligand is a modified PEG covalently bound to the inorganic surface by hydroxamic acid) to an end concentration 0.5 mg/mL was added to a final volume of 500 µL of EV solution in PBS. Incorporation of USPIO was achieved via sonication using a point sonicator in 2‐s bursts for 10 s while on ice. This cycle was repeated three times. Following sonication, another round of ExtraPEG purification was performed, and the pellet was suspended in 50 µL PBS for injection.

### 2.10 Rat Model of Ischemic Stroke

All animal procedures were completed in accordance with the Animal Care and Use Committee at the Florida State University (PROTO202000023), the United States Public Health Service's Policy on Humane Care and Use of Laboratory Animals, as well as the United States National Institutes of Health Guide for the Care and Use of Laboratory Animals. Male Sprague Dawley rats (180–225 g) underwent a middle cerebral artery occlusion (MCAO) to induce cerebral ischemia according to previous literature (Longa et al. 1989). Power analysis was performed based on preliminary studies to determine the minimal number of animals needed per group. Thirty‐one rats were randomly assigned to the following groups: hypoxia hMSCs (*n* = 8), hypoxia EVs (*n* = 12) or vehicle as control (PBS only, *n* = 11). In brief, rats were anesthetized with 4%–5% isoflurane and maintained under a surgical plane of anaesthesia after pre‐warming on a heated pad. A rubber‐coated filament (Doccol Corp., Sharon, MA) was inserted into the external carotid artery and threaded into the internal carotid artery 1.9 cm to the circle of Willis effectively occluding the middle cerebral artery. The filament was sutured in place for 1 h, and the rat allowed to regain consciousness in a heated recovery chamber. Prior to filament removal, rats were re‐anesthetized for surgery duration. The midline incision was reopened and the filament slowly removed over the course of 1 min, and the artery sutured closed. Following, a 33‐g needle with 12° bevel and Hamilton syringe was used to inject 50 µL of PBS (with or without EV or hMSC) into the ICA as a single bolus injection. The dose of hMSC group was 1 × 10^6^ cells and the EV group was the amount of EVs (8 × 10^11^) secreted by the same number of hMSCs over 2 days that went through the USPIO labelling process. To minimize potential confounders, the surgery and treatment administration was performed by the same operator throughout the study. Rats who did not develop ischemia indicated by MRI on Day 1, likely due to a misplacement of the filament, were excluded from the study.

### 2.11 MRI of Labelled hMSC‐EV *In Vivo*

All MRI experiments were performed at the 21.1‐T, 900‐MHz vertical MRI scanner at the National High Magnetic Field in Tallahassee, FL (Fu et al. 2005). The magnet was equipped with a Bruker Avance III console and scans were recorded using Paravision 5.1 (Bruker, Billerica, MA). Rats were imaged 1, 3, 7 and 21 days post‐surgery to evaluate therapeutic potential. Rats administered hypoxia EV underwent an additional, albeit brief, imaging session on Day 0 to assess EV delivery. In brief, rats were loaded into a home‐built linear birdcage double‐tuned ^23^Na/^1^H radio frequency coil tuned to 237 and 900 MHz, respectively. All rats were oriented in a supine position within the cradle and maintained at or below 3% isoflurane during imaging to ensure a steady respiration rate while in the magnet. Respiration was monitored (Small Animal Instruments, Inc., Stonybrook, NY) and used for acquisition triggering during MRI. EV biodistribution and clearance was confirmed with a ^1^H 2D GRE sequence acquired at (50‐µm) (Hong et al. 2019) in‐plane resolution using TE = 4.0 ms and TR = 1 s with one average for a total scan time of 8.5 m. Ischemic lesion localization and volumetrics were pursued utilizing T_2_‐weighted images generated by a ^1^H 2D fast spin echo (FSE) sequence with (100 µm) (Hong et al. 2019) in‐plane resolution, an effective TE = 25 ms, TR = 6 s and one average for a total scan time of 6.5 min. Ischemic lesion volumetric were established by ^23^Na MRI, which utilized a chemical shift imaging (CSI) sequence acquired at 1‐mm isotropic resolution with TR = 60 ms and 32‐min acquisition time.

Metabolite levels were measured using ^1^H relaxation‐enhanced magnetic resonance spectroscopy. Selective bandwidth excitation pulses targeted lactate, creatine, choline, and N‐acetyl aspartate, while avoiding water (Shemesh et al. 2013; Shemesh et al. 2014; Rosenberg et al. 2017). Adiabatic selective refocusing (LASER) pulses enabled spatial selectivity. T_2_W images were used for anatomical alignment of the acquired (3‐mm) (Raposo and Stoorvogel 2013) voxels in the ischemic and contralateral hemispheres.

### 2.12 MRI Data Analysis

CSI data reconstructed in MATLAB (MathWorks, Natick, MA) was zero‐filled to 0.5‐mm isotropic resolution for volumetric and signal analysis in Amira 3D Visualization Software (Thermo Fisher Scientific). A signal threshold generated from the contralateral hemisphere was used to define the ischemic lesion:

(1) $$\mathrm{Signa}l_{\mathrm{Threshold}}=\mathrm{Signa}l_{\mathrm{Contralateral}}+2.5SD_{\mathrm{Contralateral}}$$

All signals above this threshold, excluding the CSF and eyes, was assigned to the ischemic lesion and lesion volume calculated accordingly. ^23^Na signal was also determined within each ischemic lesion and the contralateral hemisphere using a comparable region of interest. For longitudinal sodium analysis, the Day 1 lesion volume was overlayed on subsequent days. ^1^H T_2_W data were reconstructed in Amira and ischemic lesion volumes calculated based also on the same threshold procedure described above. RE‐MRS data were reconstructed in JMRUI (Stefan et al. 2009) using Linear Prediction Singular Value Decomposition to select components with peaks assigned according to previous literature: NAA 2.0 ppm, Lac 1.31 ppm, Cre 3.0 ppm, Cho 3.2 ppm (Govindaraju et al. 2000), referencing water at 4.7 ppm. Metabolite ratios to choline were used to track metabolic signals longitudinally.

### 2.13 Neuromotor Assessment

Bilateral forelimb reaction was used to quantify deficit based on muscle resistance (Ishikawa et al. 2013). Rats were held vertically in a single‐handed restraint and forelimbs gently pushed in the dorsal direction. Deficits were scored 0–3 depending on resistance and return to resting position. For the beam grasp, rats were held vertical and allowed to grasp onto a ∼1.5 cm rod with scoring based on initiating grabbing and resistance with gentle pull (Ishikawa et al. 2013). The elevated body swing test (Chang et al. 2000) consisted of 20 swings in which rats were lifted vertically 15 cm above a table by the tail and immediately returned to resting position on the table following each swing. Directional preference for each swing were recorded.

### 2.14 Statistical Analysis

All statistics were performed in Prism 9.2.0 (GraphPad Software, San Diego, CA) using a mixed‐effects model with Tukey's multiple comparisons post‐hoc test. Statistical significance was defined by *p* < 0.05 with *α* = 0.05. All graphs are depicted as mean ± standard deviations.

## 3 Results

### 3.1 Transcriptome and Proteome Analysis of Hypoxia hMSCs

**FIGURE 1:**
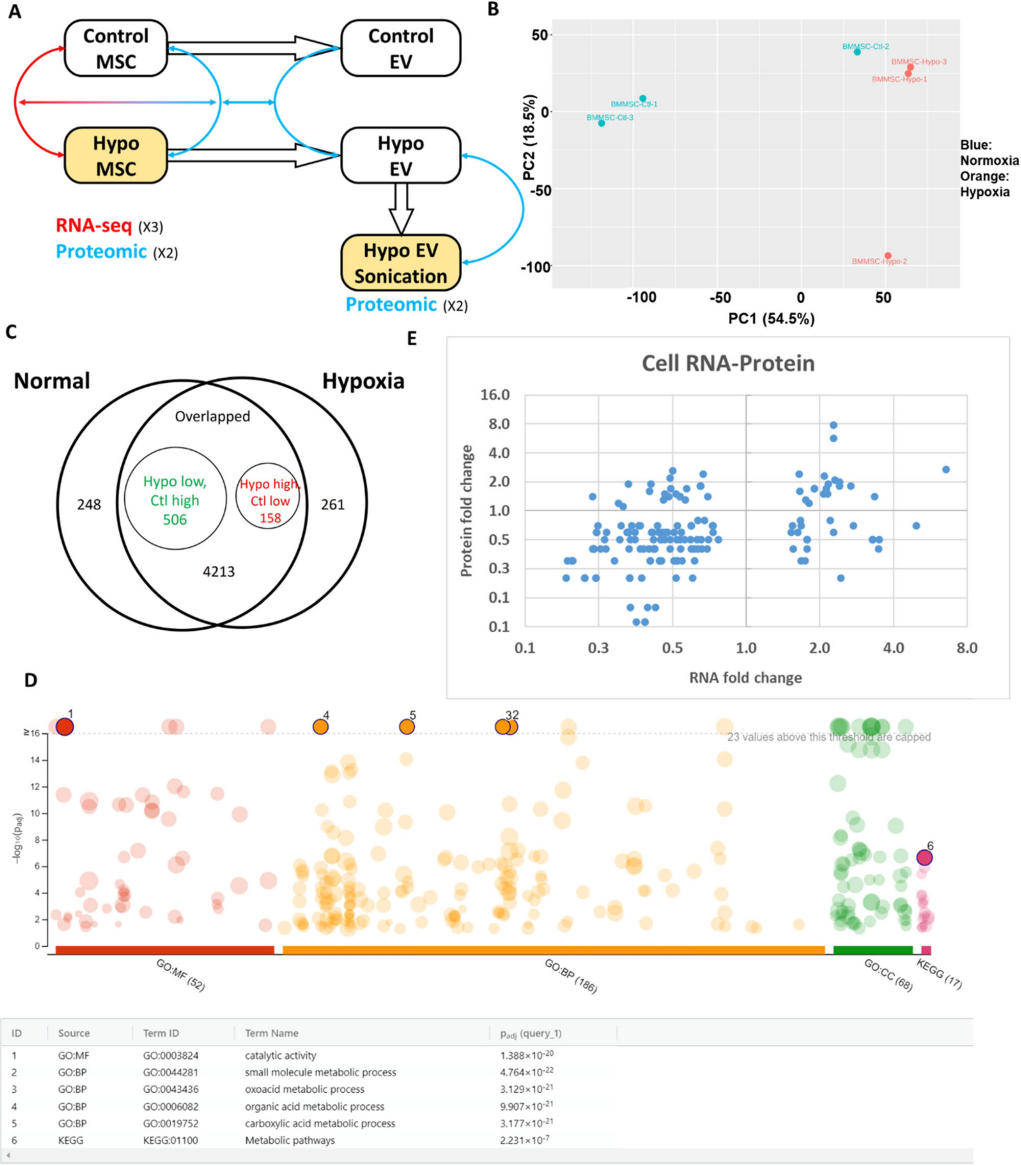
**Transcriptome and proteome analysis of hypoxia hMSCs**. (A) Schematic process flow for mRNA‐seq and proteomics analysis depicting the groups (as well as donor and technical replicate numbers) in yellow administered *in vivo*; (B) PCA plot of hypoxia and normoxia hMSC transcriptome analysis; (C) Venn diagram of hypoxia and normoxia hMSC proteomics analysis; (D) GO annotation shows the pathway analysis of DEP for hypoxia hMSCs. (E) Correlation of DEP of proteomics analysis with DEG of transcriptome analysis. For RNA‐seq, *n* = 3; for proteomics, *n* = 2 biological preparations with triplicate samples.

In vitro characterizations were performed for hypoxia hMSCs in comparison to normoxia hMSCs by mRNA‐seq (transcriptome) and proteomics (proteome) analysis (**Figure** **1A** and **Excel Data file S1, S2**). The principal component analysis (PCA) plot shows the clustering of the hypoxia and normoxia groups (three donors each) from transcriptome analysis (**Figure** **1B**). Donor‐to‐donor variations were observed, and one donor from the normoxia group was closer to the hypoxia cluster while one donor from the hypoxia group deviated. Similar observations can be seen from the heatmap (**Figure** **S1A**). The volcano plot shows the DEGs upregulated or downregulated in hypoxia versus normoxia culture with 599 upregulated and 936 downregulated at a *p* value less than 0.05 (**Figure** **S1B**). The examples of upregulated DEGs include microRNAs, *MIR4256* (3.04, logFC hypoxia vs. normoxia, FC: fold change), *MIR125B1* (2.92), *MIR324* (2.87), metabolic‐related gene *PDK1* (1.49), matrix‐related genes *COL6A6* (2.60), cell‐cycle related gene *CDKL4* (2.68), and so forth (**Data file S1**). The examples of downregulated DEGs include *MIR4785* (−3.05), *VTN* (vitronectin −2.30), *GJC2* (gap junction protein, −3.63), and so forth. EV Biogenesis genes did not seem to be affected.

The proteomics analysis showed the differentially expressed proteins (DEPs) between hypoxia and normoxia groups as seen in the Venn diagram (**Figure** **1C**). Two hundred and sixty‐one unique proteins were distinctly expressed in the hypoxia group and 248 in the normoxia group. In the total overlapping proteins, 158 were upregulated in the hypoxia group and 506 were upregulated in the normoxia group. The GO annotation confirmed that the proteins were related to metabolic pathways, carboxylic acid metabolic processes and other metabolic processes (**Figure** **1D**), showing the influence of hypoxia on hMSC metabolism. GO analysis also displayed DEPs involved in molecular functions (e.g., cell adhesion molecule binding) and cellular components (e.g., extracellular membrane‐bounded organelles and EVs) (**Table** **S2**). KEGG pathway analysis identified the top 16 pathways, which include metabolic pathways, carbon metabolism, glycolysis/gluconeogenesis, amino acid metabolism and biosynthesis of co‐factors. The plot of DEPs versus DEGs did not show a linear relationship (**Figure** **1E** and **Supplementary Excel Data file S3**), indicating that higher fold change of mRNA had weak correlation with higher fold chagne of proteins (the low and high right regions), while the lower fold change of mRNA has high likelihood to result in lower fold change of proteins (low left region) with some exceptions (high left region).

### 3.2 Characterizations of Hypoxia hMSC‐EVs and Protein Cargo by Proteomics

**FIGURE 2:**
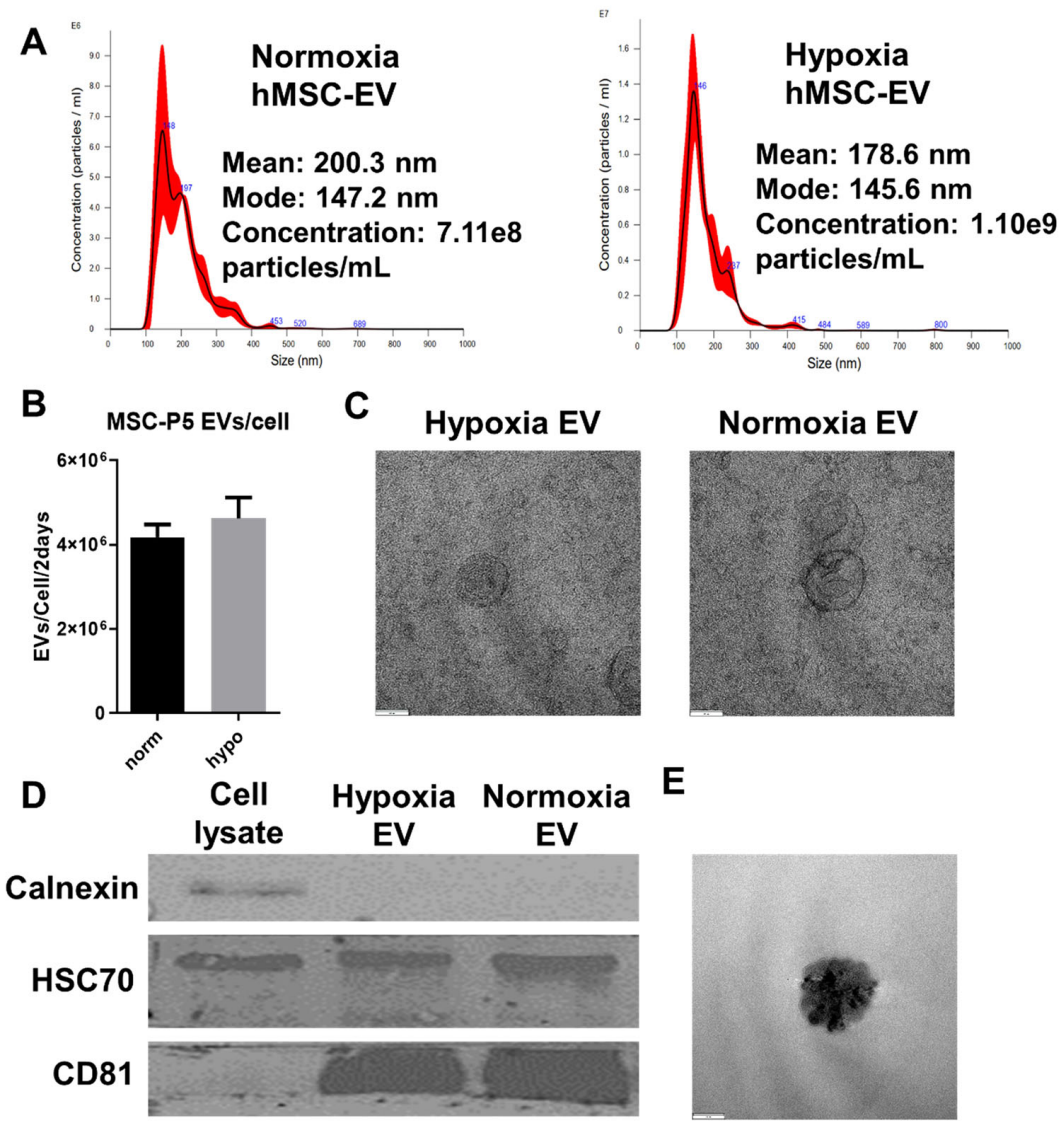
**Hypoxia hMSC‐EV characterizations**. (A) Nanoparticle tracking analysis (NTA) analysis for normoxia and hypoxia hMSC‐EV and corresponding concentration; (B) EV yield/cell/2‐day interval; (C) TEM of hypoxia and normoxia EV with 60‐nm scale bar; (D) Western blot with negative (calnexin) and positive (HSC70 and CD81) exosomal markers. (E) TEM of hypoxia EV labelled with iron oxide, with 60‐nm scale bar.

EVs were isolated from the hypoxia and normoxia hMSC‐conditioned media. Size distribution determined by NTA analysis confirmed that the mode (145 vs. 147 nm) and mean sizes (178 vs. 200 nm) were comparable, but the yield was 57% higher for the hypoxia group (0.71 vs. 1.1 × 10^9^) (**Figure** **2A**). The EV numbers were normalized to the cell number of the 2‐day culture, and the EV yield was comparable (4.0–4.5 × 10^6^ EVs/cell/2 days) for the hypoxia and the normoxia hMSCs, indicating that the increased EVs under hypoxia may be due to the increased cell number (**Figure** **2B**). Additionally, the characteristic concave EV shape was observed for both groups under TEM (**Figure** **2C**). Finally, positive (CD81, HSC70) and negative (calnexin) markers for EVs were confirmed for both EV populations by Western blot (**Figures** **2D** and **S2**). Upon iron oxide labelling, EVs maintained the nanoscale morphology (**Figure** **2E**). The total protein contents normalized to EV number were around 0.43–0.68 µg per 10^10^ EVs for both groups (**Table** **S3**).

**FIGURE 3:**
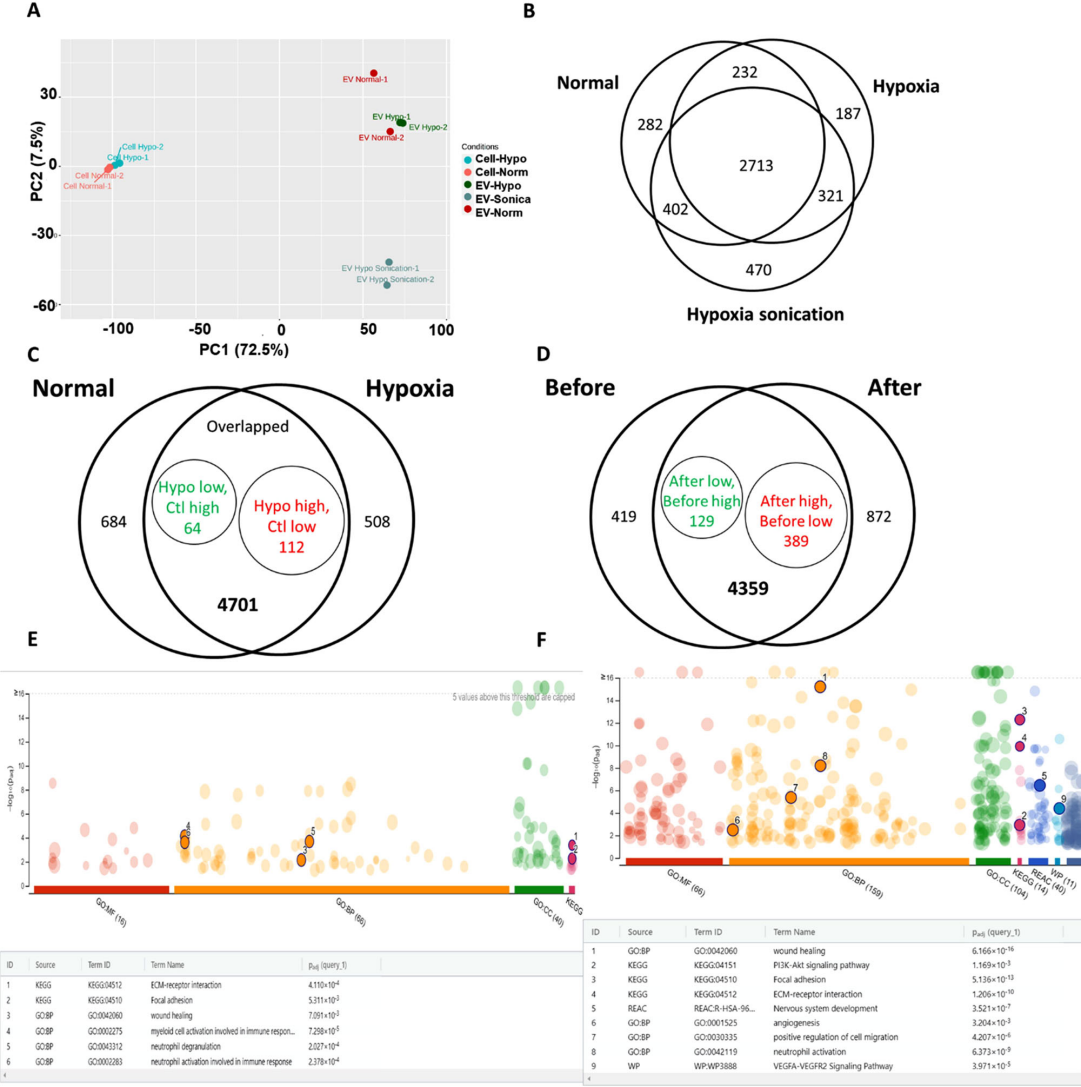
**Proteome analysis of hypoxia hMSC‐EV protein cargo**. (A) PCA plots of proteomics samples for hypoxia hMSC‐EV (before and after sonication), normoxia hMSC‐EV, as well as the corresponding parental cells (groups I‐V, respectively); (B) Venn diagram of DEP for the three EV groups; (C) Venn diagram of DEP for the comparison of hypoxia and normoxia EV; (D) Venn diagram of DEP for hypoxia EV before and after sonication; (E) GO annotation shows the pathway analysis of DEP for hypoxia hMSC‐EV; (F) GO annotation shows the pathway analysis of DEP for hypoxia hMSC‐EV after sonication.

The protein cargo of hypoxia and normoxia hMSC‐EVs was analysed by proteomics along with their parent cells (**Figure** **3** and **Supplementary Excel Data file S2**). As the hypoxia hMSC‐EVs were labelled by sonication for MRI tracking, the group of hypoxia hMSC‐EVs after sonication was included. From the PCA plot (**Figure** **3A**), the hypoxia and normoxia hMSCs were clustered together, while the hypoxia and normoxia hMSC‐EVs formed another separate cluster. Unexpectedly, the hypoxia hMSC‐EVs post‐sonication formed the third cluster, distinct from the hypoxia hMSC‐EVs before sonication. Venn diagram of the three EV groups showed a total of 4607 identified proteins, in which 2713 (58.9%) proteins were expressed by all three EV groups. Two hundred and eighty‐two (6.1%) DEPs were expressed exclusively by normoxia hMSC‐EV, 187 (4.1%) DEPs were expressed only for hypoxia hMSC‐EVs, while 470 (10.2%) DEPs were expressed exclusively by hypoxia hMSC‐EVs after sonication. Apparently, sonication has an impact on the EV protein cargo. The examples of cell/EV enriched proteins include fibronectin and filamin C (**Figure** **S3** **)**.

Additional Venn diagrams depict the influence of hypoxia on the hMSC‐EV protein cargo (**Figure** **3C**) as well as the influence of sonication on the protein cargo of hypoxia hMSC‐EVs (**Figure** **3D**). Comparing hypoxia versus normoxia hMSC‐EVs, the overlapped DEP contained 64 proteins that were low in hypoxia hMSC‐EVs, and 112 DEPs that were high in hypoxia hMSC‐EVs (**Figure** **3C**). Alternatively, 508 DEPs were evident distinctly in the hypoxia hMSC‐EVs (**Figures** **3C**, **S3 and S4**), while GO annotation confirms that these DEPs were mainly related to RNA processing and splicing. Examples of hypoxia/normoxia hMSC‐EV enriched proteins (e.g., glycogen phosphorylase) are shown in **Figure** **S5**.

Comparing the overlapped DEPs of hypoxia hMSC‐EVs before and after sonication, 129 were low in hypoxia hMSC‐EVs after sonication, and 389 DEPs were high in hypoxia hMSC‐EV after sonication while 872 DEPs were distinctly evident in hypoxia hMSC‐EVs after sonication (**Figure** **3D** and **Supplementary Excel Data file S2**). GO annotation indicates that the hypoxia hMSC‐EVs contain the altered levels of pathway proteins related to extracellular matrix (ECM)‐receptor interaction, focal adhesion, wound healing and activation of immune response (**Figure** **3E**). With respect to sonication effects, the GO annotation indicates alterations in several processes including wound healing, PI3K‐Akt signalling pathway, ECM‐receptor interaction, focal adhesion, nervous system development, angiogenesis, positive regulation of cell migration, neutrophil activation and VEGFA‐VEGFR2 signalling pathway (**Figure** **3F**).

To illustrate if the sonication effects resulted from alteration of the transmembrane protein profile, the ratio of transmembrane proteins to total proteins was analysed for hypoxia hMSC‐EVs before and after sonication (**Figure** **S6**). A similar ratio was found for the hMSC‐EVs before (26.7%) and after (20.8%) sonication, which indicates that the sonication effects are not resulted from alteration of transmembrane proteins. Examples of hypoxia hMSC‐EV sonication enriched proteins (e.g., integrin beta‐1) are shown in **Figure** **S7**.

### 3.3 Characterizations of Hypoxia hMSC‐EVs and miRNA Cargo by miRNA‐seq

**FIGURE 4:**
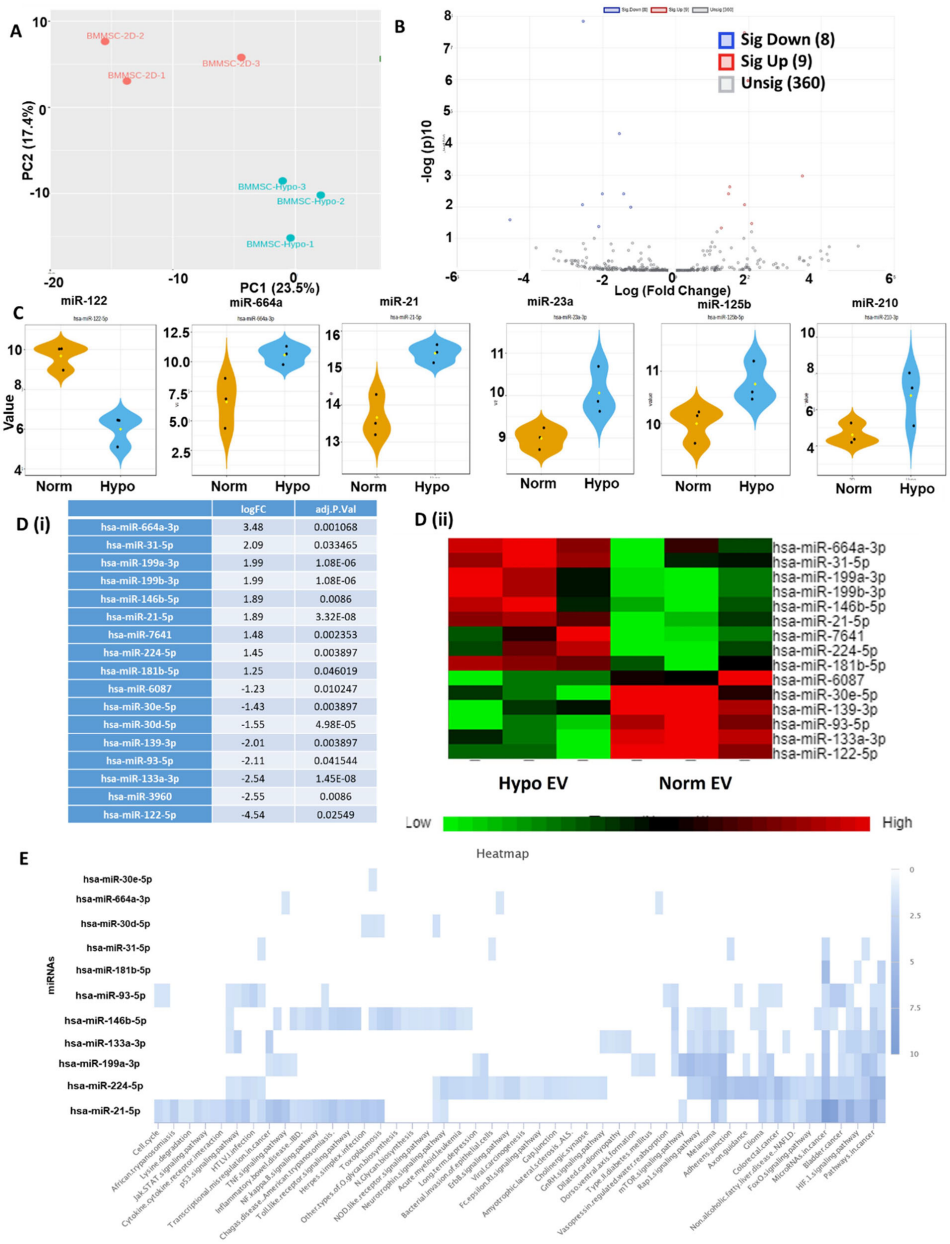
**miRNA‐sequencing analysis of hypoxia hMSC‐EV miRNA cargo**. (A) PCA plot of miRNA samples of hypoxia versus normoxia hMSC‐EVs. (B) Volcano plot of differentially expressed gene miRNAs (DEGs) of hypoxia versus normoxia hMSC‐EVs. (C) Comparison of miR‐122, 66a, 21, 23a, 125b and 210 expression. (D) (i) The list of top DEGs, positive/negative: upregulation/downregulation in hypoxia condition; (ii) Heatmap of DEG expression; (E). KEGG pathway analysis of DEG targeted genes between hypoxia versus normoxia hMSC‐EVs.

The miRNA cargo of hypoxia hMSC‐EVs was analysed against normoxia condition using miRNA‐seq (**Figure** **4** and **Table** **S4**). PCA plot showed distinct cluster between the two groups, each of which has three replicates (Figure 4A). From the volcano plot, about 377 miRNAs were identified (Figure 4B), among which nine miRNAs were significantly upregulated for hypoxia condition such as miR‐664a (log of fold change = 3.38), miR‐21 (1.89; suppresses the apoptosis of neuron cells by downregulating the expression of PTEN) (Xu et al. 2019), miR‐31 (2.09; activation of Wnt pathway, promoting proliferation and reducing apoptosis and matrix degradation (Wang et al. 2022), miR‐199a (1.99; attenuates apoptosis and inflammation) (Yu et al. 2020), miR‐199b (1.99), miR‐146b (1.89; aging process) (Dalle Carbonare et al. 2023), miR‐224 (1.45; regulating autophagy), miR‐7641 (1.45), and miR‐181 (1.25) (Figure 4C **,D,** and **Table** **S5**). Some other upregulated miRNAs were also identified, including miR‐23a (regulating immune function), miR‐125b (involved in regulating NF‐κB, p53, PI3K/Akt/mTOR, ErbB2, Wnt and other signalling pathways) (Wang et al. 2020) and miR‐210 (attenuating apoptosis). Eight miRNAs were significantly downregulated for hypoxia versus normoxia condition such as miR‐122 (−4.54) (involved in matrix metalloprotein 2 function), miR‐3960 (−2.55), miR‐133a (−2.54), miR‐93 (−2.11), miR‐139 (−2.01), miR‐30d (−1.55), miR‐30e (−1.43) and miR‐6087 (−1.23). KEGG pathway analysis of DEGs (hypoxia vs. normoxia) showed that these miRNA targeted genes are involved in HIF‐1 signalling pathway, FoXO signalling, cell cycle, pathways in cancer, axon guidance, mTOR, Rap1, NF‐kB, TNF‐a, Toll like receptor signalling pathway, p53, cytokine‐receptor signalling pathway, cell cycle and so forth (Figure 4E).

### 3.4 Ischemic Lesion and Reduced Sodium Levels Reveal Tissue Recovery

**FIGURE 5:**
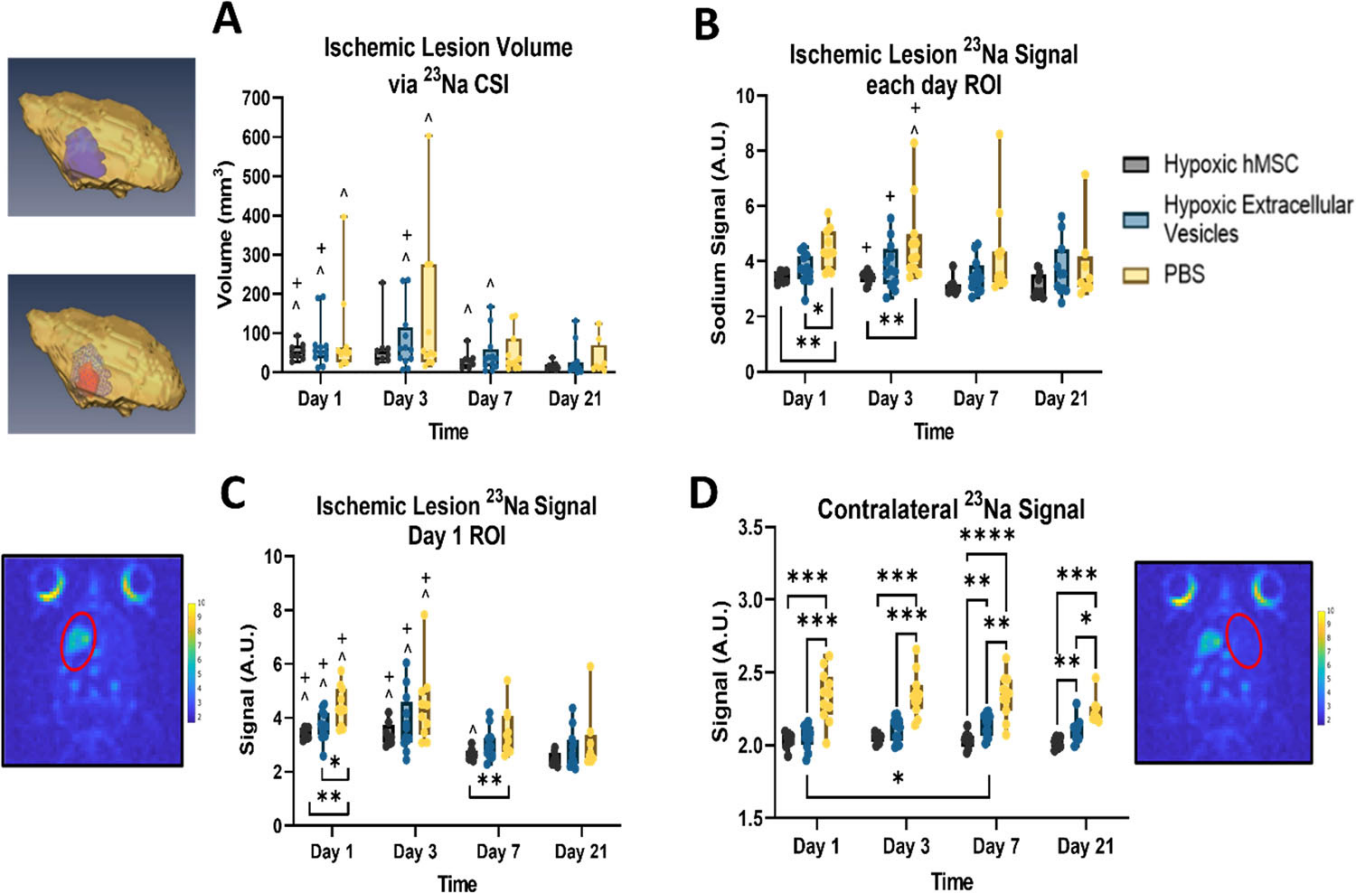
**Ischemic lesion assessment via ^23^Na MRI supports tissue recovery with hMSC‐derived therapy**. (A) Bar graph representing ischemic lesion volume and corresponding 3D rendering of a representative EV administered rat on Days 1 and 21; (B) Ischemic lesion ^23^Na signal corresponding to each day, (C) superimposed from Day 1 and (D) from the contralateral hemisphere. Statistical significance calculated using mixed effects model with Tukey's multiple comparisons test. Significance is indicated by *p* values: *p < 0.05, **p < 0.01 between hemispheres. Additionally, significance to Day 3 (#), Day 7 (+) and Day 21 (^) is marked. All values presented as mean ± SD.

Since the enhanced therapeutic benefits of hypoxia culture was well documented, our study focuses on the comparison of the therapeutic effects of hMSCs and their derived EVs to reveal the contribution from the EVs. The labelled hypoxia hMSCs and the labelled hypoxia hMSC‐EVs were administered to a preclinical model of ischemic stroke. The characterization of the labelled EVs and the initial biodistribution *in vivo* have been shown in our previous study (Helsper et al. 2024) and **Figure** **S8**. Therefore, this study focused on the effects of hMSC‐EVs on efficacy. Tissue recovery assessed by MRI/S and neuromotor improvements were compared to rats administered vehicle only. Following confirmation of therapy administration via ^1^H gradient recalled echo MRI, therapeutic efficacy was quantified on Days 1, 3, 7 and 21 post administration. Sodium MRI, specifically ^23^Na CSI, a highly sensitive metric, measured the sodium influx characteristic to cerebral ischemia in each cohort. Ischemic lesion volume as well as ^23^Na signal changes within the lesion and in the contralateral hemisphere indicated that hMSCs and the secreted EVs exhibited the increased recovery compared to vehicle animals. Volumetric analysis of the ischemic lesion between different treatment groups showed significant decreases over time but little significant difference between the groups (**Figure** **5A**). However, the distinct elevation of lesion volume from Day 1 to Day 3 in vehicle animals, particularly the high standard deviation, is typical of untreated stroke (Helsper et al. 2022). In contrast, average lesion volume in the animals injected with hMSCs or hMSC‐EVs immediately reduced by Day 3. Although all groups significantly reduced in lesion size over the 21‐day time course, rats administered hMSCs or hMSC‐EVs demonstrated faster and more significant recovery by Day 7 with continued lesion reduction to Day 21 while the vehicle cohort did not show further improvement after Day 7.

Volumetric analysis was completed utilizing ^1^H T_2_W MRI (**Figure** **S9**). Although highly valuable for visual localization of the ischemic region, typically this conventional scan is less sensitive than ^23^Na CSI. Nonetheless, trends and significance were comparable to those assessed via ^23^Na CSI. Primarily, rats administered with hMSCs exhibited an earlier reduction in total ischemic lesion volume and more significant change in volume compared to vehicle animals.

Sodium signal intensity within the ischemic lesion and in the contralateral hemisphere were assessed. As seen in **Figure** **5B**, average ^23^Na signal of the ischemic lesion was elevated in the vehicle group compared to both hMSC and hMSC‐EV administered rats. This significance remained on Day 3 for the hMSC group. Although signal did reduce in the vehicle cohort over the 21‐day time course, the early return toward sodium baseline levels in hMSC and hMSC‐EV administered rats demonstrated a distinct level of recovery. An alternative analysis to capture effects of sodium level within the recovering tissue also was pursued. In brief, the ischemic lesion volume on Day 1 was superimposed on subsequent days. As the lesion reduced over time, the volume defined on Day 1 thus captured the salvageable region of tissue surrounding the ischemic core. **Figure** **5C** demonstrates that while longitudinal trends remained comparable to the previous method, significance between treatment groups emerged on Day 7 between hMSC and vehicle groups. hMSCs generated an early response for tissue recovery compared to controls while hMSC‐EVs provoked more of an intermediate response.

Systemic effects of the two treatments were evaluated in the contralateral hemisphere by assessing ^23^Na signal. As can be clearly seen in **Figure** **5D**, both hMSC and hMSC‐EV groups maintained significantly lower ^23^Na signal than the PBS groups at every time point. This phenomenon has been described previously, whereas a successful therapy establishes the maintenance of low sodium levels in the contralateral hemisphere compared to vehicle animals, or even unsuccessful therapy (Helsper et al. 2023). This level of significance between the hMSC‐EV and vehicle groups is strong evidence of the therapeutic potential exhibited by the hMSC secretome, delivered as hMSC‐EVs.

### 3.5 Metabolic Assessment Indicates Energetic Recovery

**FIGURE 6:**
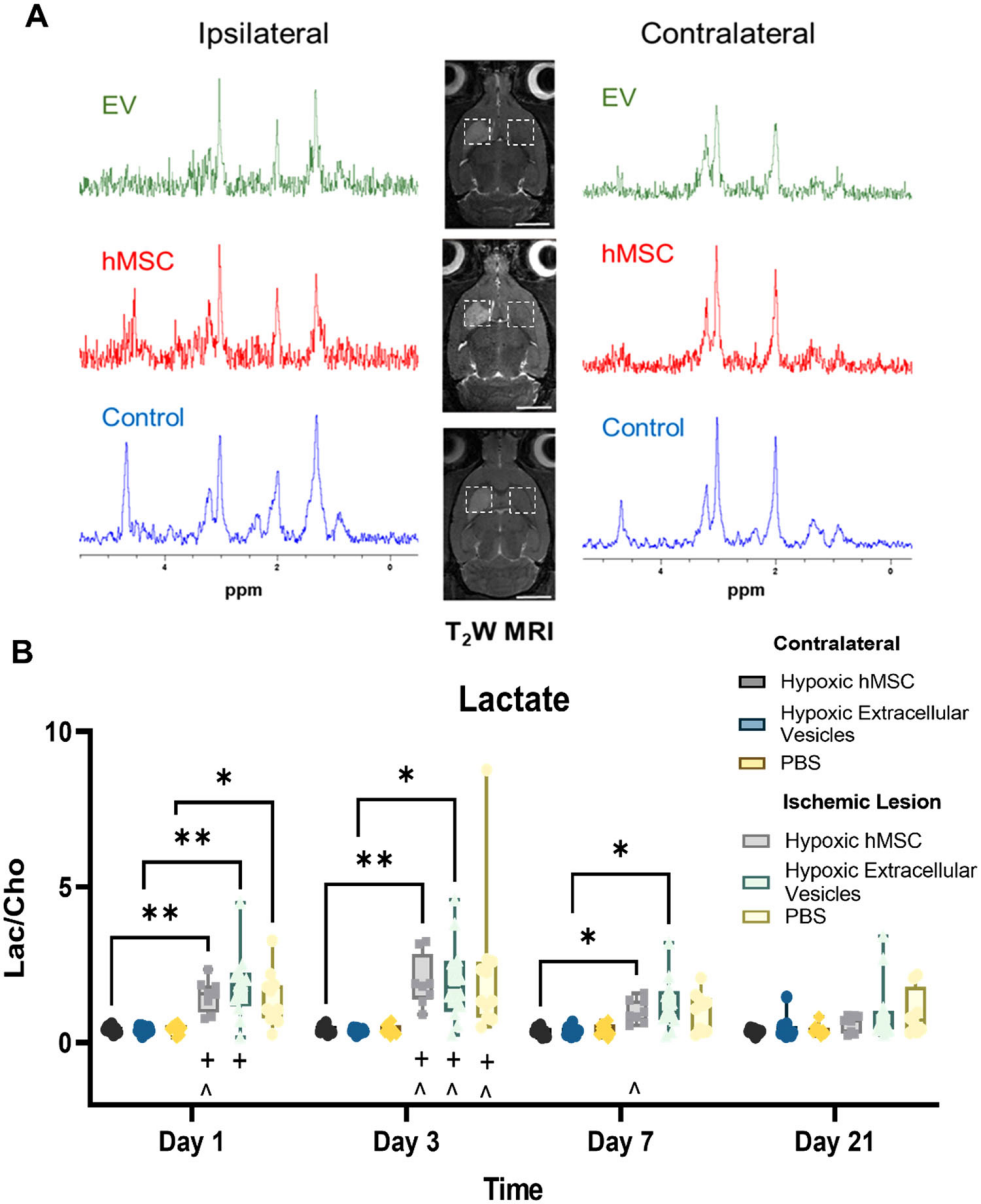
**^1^H MRS supports energetic recovery following administration of hypoxia hMSC‐derived therapy**. (A) Representative ^1^H MRS 1‐d post MCAO in the ipsilateral and contralateral hemispheres; (B) Bar graph representing lactate to choline metabolic ratios for inter‐ and intra‐group comparisons. Statistical significance calculated using mixed effects model with Tukey's multiple comparisons test. Significance is indicated by *p* values: *p < 0.05, **p < 0.01 between hemispheres. Additionally, significance to Day 3 (#), Day 7 (+) and Day 21 (^) is marked. All values presented as mean ± SD.

Metabolic changes, particularly lactate levels, provide insight into cerebral energetics within defined regions. Hemispherical differences within a treatment group as well as changes between treatment groups for lactate levels are demonstrated in **Figure** **6**. As expected, all three groups exhibited significant lactate elevation in the ischemic region compared to the contralateral hemisphere. High standard deviation in the vehicle group lowered significance at later time points. However, when longitudinal changes were evaluated to determine recovery within each hemisphere, notably, the lactate levels recovered for hypoxia‐hMSC animals on Day 7 while the hypoxia‐EV group recovered by Day 21. In contrast, the PBS group exhibited prolonged elevation of lactate throughout the entire time course indicating limited ability to reestablish energetic levels following an ischemic stroke without a therapy onboard.

### 3.6 Recovery of Neuromotor Activity and Behavior Improvements

**FIGURE 7:**
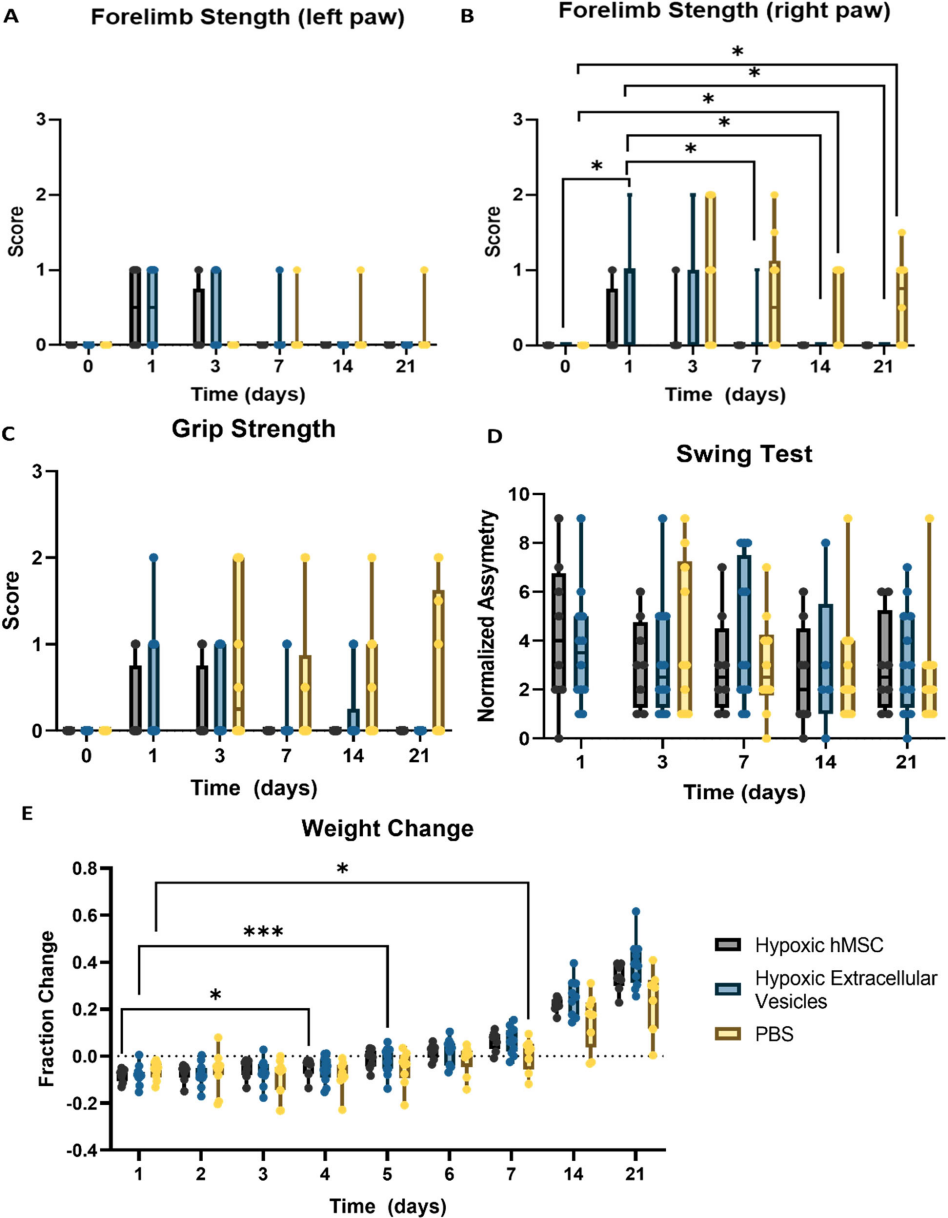
**Neuromotor assessment of rats following MCAO and therapy administration**. Bar graphs representing (A) forelimb strength in left and (B) right front paws, (C) grip strength, (D) asymmetry scores, and (E) weight change following MCAO throughout the 21‐day time course. Scores were assigned 0–3. Statistical significance was calculated using mixed effects model with Tukey's multiple comparisons test. Significance is indicated by *p* values: *p < 0.05. All values presented as mean ± SD.

Functional recovery following MCAO was monitored via neuromotor assessments. Previous studies have demonstrated hMSC release neurotrophic factors that are integral to neural circuit development and can be upregulated by hypoxia (Teixeira et al. 2013). One method of assessing neural recovery is through functional behaviour. Forearm strength tests were performed on both front paws to probe irregularities over time and between treatment groups (**Figure** **7A**,**B**). Forelimb strength was assigned a score 0–3, with 0 indicative of no impairment and 3 severe impairments when slight pressure was applied to the footpad toward the dorsal direction. As expected, the left paw exhibited minimal strength impairment over the entire time course with a few rats exhibiting slight increases on Days 1 and 3. However, the right forelimb exhibited significantly increased impairment from baseline for hMSC‐EV administered rats on Day 1 and PBS administered rats on Day 3. Hypoxia hMSC‐EV animals were able to quickly regain their forelimb strength by Day 7 while PBS animals exhibited lasting effects through Day 21.

In tandem to forelimb, grip strength was assessed by scoring the ability for rats to hold on to a rod with both front paws (**Figure** **7C**). Scores were again marked 0 (no difficulty) to 3 (unable to grasp with either forepaw). Although trends follow those seen in the forelimb strength test, significance was not established. Nevertheless, vehicle animals were the only group unable to regain full grip strength by Day 21. Asymmetry was assessed via the elevated body swing test by gently lifting the rats off a level surface and marking their preferred direction determined by a 10° shift from neutral in head position. All scores were normalized to baseline (pre‐surgery) to alleviate specific directional preference. Although trends indicated slight recovery over time, groups exhibited comparable asymmetry scores (**Figure** **7D**).

In addition to functional recovery, weight was monitored for all rats on Days 1 through 7, 14 and 21. Weights were noted prior to imaging sessions, and fractional change was normalized to pre‐surgery Day 0 measurements. As demonstrated in **Figure** **7E**, rats administered hypoxia‐hMSC exhibited significant weight gain between Days 1 and 4 while hypoxia hMSC‐EV generated a robust recovery by Day 5. In contrast, vehicle animals had a severely delayed weight gain reaching significance only on Day 7 with consistently lower weight gain at Days 14 and 21. This finding supports the earlier assessments that, although not as robust as hMSC directly, hMSC‐EV were able to generate a therapeutic response not seen in vehicle‐administered animals.

### 3.7 Correlation of Proteome Analysis of Hypoxia hMSC‐EVs With *In Vivo* Outcome

The therapeutic benefits of hMSCs have been reported as the abilities of immunomodulation, neuroprotection and secretion of neurotrophic factors, as well as promoting angiogenesis, neurogenesis and cell migration (Kurozumi et al. 2005; Zheng et al. 2018; Wang et al. 2011). Hypoxia preconditioning on hMSCs enhance the therapeutic effects due to the upregulation of hypoxia‐inducible factor (HIF) signalling and the hypoxia hMSC‐EVs should carry the therapeutic protein cargo. Proteomics data show that hypoxia hMSCs have high expression of metabolic proteins related to HIF signalling (**Figure** **S10**) such as SLC2A1 and LDHA (**Table** **1**). The secreted hypoxia hMSC‐EVs after sonication also upregulated the expression of SLC2A1 and LDHA. In terms of EV biogenesis, hypoxia hMSC‐EVs after sonication show high expression of CD81, Alix, Rab27B and TSG101. The hypoxia hMSCs increased CD81 but reduced Alix (ESCRT‐dependent) expression. For neurogenesis, hypoxia hMSCs have higher levels of TGM2 and APPL2 than the hypoxia hMSC‐EVs after sonication, while the EV samples have high expression of NAP1L1 and DLL4.

**TABLE 1:** Identified proteins related with HIF, EV biogenesis and neurogenesis pathways.

Pathways	Protein ID	Cell Hypoxia versus normoxia		EV After versus before sonication	
		*T*‐test (*p* value)	Fold change	*T*‐test (*p* value)	Fold change
HIF pathway	VEGFC	0.61	2.5	0.12	0.0
	EGFR	0.14	0.6	0.14	INF
	ANGPT1	1.00	INF	0.57	0.3
	TIMP	0.62	1.1	<0.00010	0.0
	SLC2A1	0.02	2.3	0.01	2.4
	GAPDH	0.13	1.3	0.45	0.8
	LDHA	0.01	2.1	0.01	1.7
EV biogenesis	CD81	0.17	1.9	0.00	2.3
	PDCD6IP	0.03	0.6	0.04	1.0
	Rab27B	1.00	INF	0.03	12.0
	VIM	0.06	1.3	0.17	0.2
	TSG101	0.49	0.8	0.02	5.5
Positive regulation of neurogenesis	PEDF	0.24	0.0	0.08	0.5
	TGM2	0.57	0.9	0.14	1.9
	NAP1L1	0.49	0.8	0.10	1.3
	NAV3	0.11	4.1	0.82	1.5
	DLL4	0.60	0.3	0.91	1.1
Negative regulation of neurogenesis	APPL2	0.03	0.5	0.42	INF

*Note*: Red in the *t* test column indicates *p* value < 0.05; Red and green in the fold change column indicates up‐ or down‐regulated, respectively.

For the proteins related to angiogenesis‐sprouting (**Table** **2**), hypoxia hMSCs have high expression of EPHA2, ITGB1, CDH13 and NRP1, while the sonicated hMSC‐EV samples have high expression of LOXL2, SLIT2, RAC1 and THBS1. For the proteins related to angiogenesis‐positive regulation, the hypoxia hMSCs have high expression of RRAS, ITGB1 and NRP1, while the sonicated hMSC‐EV samples have high expression of C3, C5, CDKL2, SERPINE1, PDCD6, THBS1 and WNT5A. For the proteins related to angiogenesis‐negative regulation (**Table** **S6**), the hypoxia hMSCs have high expression of CTNNB1 and SPARC, while the sonicated hMSC‐EV samples have high expression of HSPG2, AMOT, ROCK1, ROCK2 and STAB1. Proteins related to cell migration (**Table** **3**) were analysed and the results indicated that the hypoxia hMSCs exhibited high expression of ITGB1 and EPHA2 while the sonicated hMSC‐EV samples have high expression of PDGFC as well as ITFB1 compared to the other EV samples. These DEPs in the hMSC‐EVs and hMSCs may contribute to the *in vivo* functional outcome compared to the vehicle only group.

**TABLE 2:** Identified proteins related with angiogenesis pathways.

Pathways	Protein ID	Cell Hypoxia versus normoxia		EV After versus before sonication	
		*T* test (*p* value)	Fold change	*T*‐test (*p* value)	Fold change
Sprouting angiogenesis	AKT1	0.079	0.20	0.98	1
	LOXL2	0.031	2.10	0.0052	0.7
	SLIT2	0.270	3.80	0.17	0.4
	EPHA2	0.008	1.40	0.091	2.2
	ITGB1	0.160	1.20	0.0027	1.9
	RAC1	0.110	1.30	0.012	2.2
	GPLD1	0.420	0.00	0.16	0.5
	CDH13	0.160	1.10	0.91	1
	THBS1	0.033	1.80	0.0051	0.6
	NRP1	0.660	0.80	0.065	INF
Positive regulation of angiogenesis	ANGPTL4	1.000	INF	0.59	1.3
	C3	0.350	0.20	0.011	0.7
	C5	0.160	INF	0.67	1.1
	ENG	0.400	1.30	0.00099	2.6
	CDKL2	0.130	0.20	0.35	1.4
	HSPB1	0.640	1.10	0.35	1.3
	SERPINE1	0.330	0.90	0.25	1.1
	PDCD6	0.530	1.20	0.077	1.5
	RRAS	0.330	1.10	0.028	3.3
	THBS1	0.033	1.80	0.0051	0.6
	STAT3	0.160	0.40	0.032	0.5
	TERT	0.420	0.00	0.42	INF
	ITGB1	0.160	1.20	0.0027	1.9
	WNT5A	0.770	0.90	0.39	0.8
	NRP1	0.660	0.80	0.065	INF

*Note*: Red in the *t*‐test column indicates *p* value < 0.05; Red and green in the fold change column indicates up‐ or down‐regulated, respectively.

**TABLE 3:** Identified proteins related with migration pathways.

Pathways	Protein ID	Cell Hypoxia versus normoxia		EV After versus before sonication	
		*T*‐test (*p* value)	Fold change	*T* test (*p* value)	Fold change
Cell migration involved in sprouting angiogenesis	EPHA2	0.008	1.40	0.091	2.2
	AKT1	0.079	0.20	0.98	1
	ITGB1	0.160	1.20	0.0027	1.9
	SLIT2	0.270	3.80	0.17	0.4
	GPLD1	0.420	0.00	0.16	0.5
	NRP1	0.660	0.80	0.065	INF
Mesenchymal stem cell migration and tissue repair	TGFBI	0.006	2.20	0.019	0.6
	FGF2	0.910	1.00	1	INF
	VEGFC	0.610	2.50	1	INF
	VEGFD	0.540	0.20	0.12	0
	PDGFC	1.000	INF	0.38	0.8
	ANGPT1	1.000	INF	0.57	0.3

*Note*: Red in the *t*‐test column indicates *p* value < 0.05; Red and green in the fold change column indicates up‐ or down‐regulated, respectively.

## 4 Discussion

The therapeutic efficacy of hMSCs preconditioned *via* hypoxia and hMSC‐EVs following equivalent preconditioning was assessed *in vitro* and *in vivo*. The goal of this study was to investigate the contribution of hypoxia hMSC‐EVs to tissue recovery following ischemia in stroke and the therapeutic response compared to the parent cells. Due to the increased therapeutic benefits of hypoxia preconditioning (Yuan et al. 2019), the proteomics of hypoxia hMSCs and their derived EVs for protein cargo before and after sonication were compared. The miRNA cargo of hMSC‐EVs influenced by hypoxia was also revealed. Compared to their parent cells, EVs induce lower immune responses, are less prone to damage during delivery and more drug‐like, and do not have uncontrolled proliferation or differentiation.

Endogenously, bone‐marrow derived hMSCs reside in a low oxygen tension niche, equivalent to 1%–2% O_2_ when measured in mice (Spencer et al. 2014); thus, culturing hMSCs under hypoxia recapitulates the innate environment from which they are derived leading to many therapeutic benefits (Liu et al. 2017). Preconditioning of hMSC culture indeed has displayed enhanced therapeutic effects in several disease models including skeletal muscle regeneration (Archacka et al. 2021) and intracerebral haemorrhage (Liu et al. 2021). Similar to bone marrow derived hMSCs, adipose hMSCs also have demonstrated effects on functional properties and therapeutic enhancements from hypoxia conditioning (Choi et al. 2014). The benefits of hypoxia hMSCs have been investigated in previous studies (Rosenberg et al. 2013). Hypoxia hMSCs upregulate HIF signalling, promote glycolysis metabolism and secretion of neuroprotective factors, as well as promote the primitive phenotype of the stem cells (Wei et al. 2012).

As an extension, EVs derived from hypoxia hMSCs have displayed increased therapeutic advantages (Zhu et al. 2018; Wang et al. 2022). In fact, hypoxia (5% O_2_) hMSC‐EVs were shown to increase vascular tube formation *in vitro* compared to normoxia (21% O_2_) EVs (Almeria et al. 2019). These studies, and others involving alternative preconditioning methods such as 3D aggregation of hMSCs (Yuan et al. 2022; Cone et al. 2021; Liu et al. 2023), suggest that cellular alterations resulting from culture environment are carried over into their secreted EVs. Therefore, hMSC‐EVs were assessed to investigate the impact of culture conditions on the EV cargo profile. Proteomic analysis of EV protein cargo was performed on the three EV groups: normoxia, hypoxia and hypoxia after sonication. For the hypoxia‐cultured hMSCs, the most altered pathways were associated with catalytic activity and metabolism. The influence of hypoxia on MSC EV protein cargo indicated the enrichment of pathways and biological processes related to glycolysis, the immune system and ECM organization (Braga et al. 2022; Bister et al. 2020). Our study showed consistent results in line with these studies. However, none of these studies have performed proteomic analysis on USPIO labelled hMSC‐EVs to investigate how labelling may be impacting their therapeutic efficacy. In addition, the role of microRNA cargo in the EVs was evaluated using miRNA‐seq in this study. Hypoxia hMSC‐EVs showed the upregulation of miR‐664a, 21, 31, 125, 146b, 210, 199a, 199b, 224, and so forth. These miRNAs play a key role in promoting cell proliferation (cell cycle modulation), angiogenesis, reducing apoptosis, modulating immune functions through upregulation of HIF pathway, Wnt/beta‐catenin pathway, as well as modulating p53, PI3K/Akt/mTOR, ErbB2 signalling and so forth (Xu et al. 2019; Wang et al. 2022; Yu et al. 2020; Dalle Carbonare et al. 2023; Wang et al. 2020). The function in reducing matrix degradation, regulating cytokine and its receptor interaction, and regulating axon guidance and so forth was also identified. For the specific contribution of these microRNAs in the EV cargo to stroke recovery, there is much less understanding in the literature. One possible influence is miR‐21 expression (Bister et al. 2020), as indicated in this study. The potential cargo modification (e.g., using 3D aggregated hMSC‐EVs [Yuan et al. 2022; Liu et al. 2023; Jeske et al. 2023]) or the overexpression/loading of the contributing therapeutic proteins or microRNA may elevate the therapeutic outcome of hMSC‐EV based therapies (Jeske et al. 2023; Park et al. 2019).

hMSC‐EVs were labelled with an MRI sensitive contrast agent to verify administration immediately following MCAO and recirculation. However, sonication, a necessary step to incorporate the MRI visible contrast agent with the EVs, has the potential to alter EV cargo content, and thus the impact of EV sonication was evaluated *in vitro* for this study. To label hMSC‐EVs, a modified approach utilizing polyethylene glycol harvesting and purification was used in conjunction with short bursts of sonication. This method allows for the USPIO nanoparticles to be incorporated with the EVs without significantly affecting their exosome‐specific protein markers, size and morphology. As the sonication may affect therapeutic efficacy when applied *in vivo*, global proteomics was performed to characterize the EV cargo. Sonication resulted in approximately twice the distinct proteins detected only in sonicated EVs compared to un‐sonicated EVs following hypoxia conditioning. Additional assessment evaluated the effect of sonication on transmembrane proteins, showing a comparable ratio of total protein to un‐sonicated group. Previously published literature proved that sonication would alter the size, protein/miR cargo, lipids and other contents of exosomes (Nizamudeen et al. 2021; Kim et al. 2016; Liu et al. 2022; Luan et al. 2017), possibly due to membrane disruption and protein exchange. The mechanical shear force from the sonicator probe compromises the membrane integrity of the EVs and allows the USPIO to diffuse into EVs during membrane deformation. Nevertheless, this membrane deformation process does not significantly affect the membrane‐bound proteins. Haney et al. have reported that sonication and extrusion provide the highest catalase loading into exosomes, as compared with freeze/thaw cycles and passive incubation, which yield the lowest drug loading efficiency (Haney et al. 2015). In our dataset, the top 20 proteins were quite similar, and most of the un‐overlapped proteins are located in cytosol or cytoplasm, as expected.

This study focused on the utilization of sonication to load the iron oxide nanoparticles into the hMSC‐EVs; however, different iron oxide loading methods such as electroporation may be investigated (Hood et al. 2013; Hu et al. 2015). The labelling efficiency was mainly reflected by the iron content in the EVs, measured by Inductively coupled plasma mass spectroscopy (ICP‐MS). For example, the unlabelled hMSC EVs contained 1.3×10^−10^ ng Fe per EV, and the labelled EVs contained 2.6×10^−10^ ng Fe per EV, based on our preliminary results. During 21 days after administration, the labelled EVs should be taken by the cells. USPIO and the iron content went through the cellular iron metabolic system and had minimal cytotoxicity.

The therapeutic efficacy of both hypoxia hMSCs and hMSC‐EVs after sonication were evaluated *in vivo*. MRI is routinely used in the clinic to evaluate incoming stroke patients with several imaging sequences available (Burgess and Kidwell 2011). Recently, ^23^Na MRI has become feasible in the assessment of such patients, enabling detection of endogenous bulk sodium to quantify tissue sodium content in both affected and healthy tissue (Helsper et al. 2022, 2023). Changes in tissue sodium, primarily the influx of sodium into the ischemic lesion, results from the malfunction of the Na^+^/K^+^‐pump. During an ischemic event, oxygen and glucose significantly decrease as blood supply is restricted, ultimately resulting in a cellular energy metabolism crisis (Wetterling et al. 2015). 3D ^23^Na chemical shift imaging MRI enables detection of bulk sodium and provides a platform to delineate the ischemic lesion from healthy tissue in a more sensitive manner than conventional ^1^H T_2_W MRI (Helsper et al. 2022, 2023). When applied in this study, sodium metrics confirmed that the administration of hypoxia hMSCs and hypoxia hMSC‐EVs were able to assist in cerebral tissue recovery, particularly in restoring ionic homeostasis within the ischemic lesion. When Day 1 volumetrics were overlaid on subsequent days, salvageable tissue surrounding the infarct also demonstrated significant reduction in bulk sodium. To evaluate systemic effects, sodium levels in the contralateral hemisphere was evaluated. The two treated groups, hypoxia hMSCs and hypoxia hMSC‐EVs maintained low contralateral sodium signal levels in contrast to vehicle administered rats, comparable to that previously seen in hMSCs generating a robust therapeutic response (Helsper et al. 2023).

A secondary confirmation of longitudinal tissue recovery *in vivo* was investigated via ^1^H MRS, which non‐invasively measures select metabolite levels in the brain (Shemesh et al. 2013; Rosenberg et al. 2017). Lactate, a marker for anaerobic metabolism, rapidly elevates in ischemic tissue, and studies have suggested lactate to choline ratios may assist in clinical settings. Here, lactate levels increased in all groups, as expected, at early time points. Over time, the hypoxia hMSC‐administered cohort exhibited a reduction in lactate levels followed shortly after by the hypoxia hMSC‐EV administered cohort. Although not as strongly correlated as the sodium measurements, lactate recovery supports the therapeutic efficacy of hypoxia hMSCs. Promisingly, hypoxia hMSC‐EVs were able to recover a level of energetic homeostasis, indicating that hypoxia hMSC‐derived EVs have therapeutic capabilities and were able to influence endogenous recovery activity.

Although only single bolus injections of hMSC‐EVs were administered for each animal, future studies should evaluate the potential of increased EV content and/or injections. This study utilized the EVs secreted from ∼1 million hMSCs in culture over 3 days in order to equate the number of EVs that are estimated to be secreted from hMSCs *in vivo*. As our previous study indicates a clearance of approximately 80% hMSCs after 3 days of injection, this quantity of EVs should be nearly equivalent (Helsper et al. 2023). As a foundational study, this minimal EV number has been used as the baseline of therapy required to initiate a therapeutic response; however, the dose effect of hMSC‐EVs has been recognized and needs further investigation (Otero‐Ortega et al. 2020). One of the advantages of hMSC‐EVs include their small size, making them good candidates for intravenous injections that can easily be accomplished over several injection time periods, though systemic hMSC‐EVs losses are anticipated. Indeed, hMSC‐EVs have been used successfully in the clinic using a multi‐bolus approach (Kordelas et al. 2014). The applications of hMSC‐EVs in clinical trials require the understanding of EV long‐term biodistribution and correlating the EV cargo with the therapeutic effects, which motivated this study to label the EVs with USPIO and track the injected EVs using MRI. Evaluating the ability of EVs to penetrate the blood–brain barrier and the tracking EV uptake (e.g., clathrin‐mediated uptake, phagocytosis, etc.) by the recipient cells *in vivo* are important in future work. Moreover, the large‐scale EV labelling process may need to be developed. As the ultracentrifugation process is not easily scaled up to isolate the labelled EVs, tangential filtration flow method or size chromatography may need to be established. The stability of the EVs can be significantly improved using the EV lyophilization method. The future use of hMSC‐EVs in clinical trials still need to address the challenges of EV biomanufacturing as a human body would require trillion cells to harvest EVs that are sufficient for one dose injection. The defined EV cargo profiles such as the overexpression of specific mechanism contributing miRNAs (e.g., loading miR‐21 into the EVs), and the EV route administrations such as intranasal injection need to be investigated in the future (Helsper et al. 2024; Welsh et al. 2024).

## 5 Conclusion

Hypoxia preconditioning has been shown to upregulate many therapeutically beneficial pathways in hMSCs, which were extended into their secretome in the form of secreted EVs. Furthermore, although un‐sonicated hypoxia hMSC‐EVs demonstrated proteomic deviation from sonicated hypoxia hMSC‐EVs, therapeutic efficacy was still established *in vivo*. Functional recovery of the rats supports the findings following ^23^Na MRI and ^1^H MRS in terms of ionic and energetic homeostasis recovery, respectively. Therefore, the direct comparison of hMSCs and their derived EVs in a preclinical model of ischemic stroke, as demonstrated here, indicates the mechanism of recovery that is instituted with a successful therapy onboard relies, at least in part, on the hMSC secretome rather than the cells themselves.

## Author Contributions

**Shannon Helsper**: Conceptualization; data curation; formal analysis; investigation; methodology; visualization; writing–original draft; writing–review and editing. **Li Sun**: Data curation; formal analysis; investigation; methodology; software; validation; writing–original draft; writing–review and editing. **Chang Liu**: Data curation; investigation; methodology; validation. **Jacob Athey**: Formal analysis; investigation; methodology. **Yan Li**: Conceptualization; data curation; funding acquisition; investigation; methodology; project administration; resources; supervision; visualization; writing–original draft; writing–review and editing. **Richard Jeske**: Data curation; Formal analysis; Investigation; Methodology; Validation; Writing–review and editing. **Xuegang Yuan**: Conceptualization; Data curation; Formal analysis; Investigation; Methodology; Validation. **Samuel Colles Grant**: Conceptualization; Funding acquisition; Investigation; Methodology; Project administration; Resources; Supervision; Visualization; Writing–review and editing

## Ethics Statement

All applicable international, national and/or institutional guidelines for the care and use of animals were followed. All animal procedures were completed in accordance with the Animal Care and Use Committee at the Florida State University (Animal Welfare Assurance number D16‐00491) under protocol number 202000023 (Cellular‐Derived Therapy in the Treatment of Stroke, approval 07/01/2020). Additionally, all studies were conducted in accordance with the United States Public Health Service's Policy on Humane Care and Use of Laboratory Animals as well as the US NIH Guide for the Care and Use of Laboratory Animals (NIH Publications, No. 8023, 1978 revision).

## Consent

All authors have consent for publication.

## Conflicts of Interest

The authors declare no competing interests.

## Supporting information




**Supplemental Data files (excel) S1**. DEGs of hypoxia and normoxia hMSCs from mRNA‐Seq.

**Supplemental Data files (excel) S2**. Proteomics data of hMSCs and the secreted EVs.

**Supplemental Data files (excel) S3**. Correlation of mRNA with the cell protein (RNA‐Protein).

**Supplemental Figure S1**. mRNA‐seq analysis of hypoxia hMSCs.
**Supplemental Figure S2**. Original Western blot image for Figure 2D.
**Supplemental Figure S3**. Examples of proteins differentially expressed in hMSCs and the secreted EVs: Cell/EV enriched proteins.
**Supplemental Figure S4**. GO annotation of EV‐hypoxia only proteins (508) by proteomics.
**Supplemental Figure S5**. Examples of proteins differentially expressed in hypoxia EVs and the normoxia EVs: EV (hypoxia/normoxia) enriched proteins.
**Supplemental Figure S6**. Analysis of transmembrane protein before and after EV sonication.
**Supplemental Figure S7**. Examples of proteins differentially expressed in hypoxia EVs before and after sonication: EV (before/after sonication) enriched proteins.
**Supplemental Figure S8**. MRI to assess the distribution of labelled EVs and hMSCs at the ischemic lesion site.
**Supplemental Figure S9**. ^1^H MRI analysis of hypoxia hMSC and EV treated rats following MCAO.
**Supplemental Figure S10**. Pathway analysis of proteins related to HIF signaling.
**Supplemental Table S1**. Donor information of bone marrow derived hMSCs.
**Supplemental Table S2**. GO analysis (top) and KEGG analysis (bottom) for hypoxia versus normoxia hMSC proteomics datasets.
**Supplemental Table S3**. The NTA result and protein quantification of each type of EVs.
**Supplemental Table S4**. The miR and library quantification of each type of EVs.
**Supplemental Table S5**. A list of mostly upregulated or downregulated miRNAs in the EVs.
**Supplemental Table S6**. A list of proteins related to negative regulation of angiogenesis.

## Data Availability

The datasets generated during and/or analysed during the current study are available from the corresponding authors on reasonable request. The RNA‐seq data have been deposited in NCBI's Gene Expression Omnibus and are accessible through GEO Series GSE229825. The mass spectrometry proteomics data have been deposited to the ProteomeXchange Consortium *via* the PRIDE partner repository with the dataset identifier PXD041677 an https://doi.org/10.6019/PXD041677.
