# Supplementary material for: Cargo Analysis and MRI‐Based Therapeutic Assessment of Iron Oxide Labelled Extracellular Vesicles of Hypoxia Human Stem Cells in Ischemic Stroke
Source: J Extracell Biol. 2025 Jul 17;4(7):e70063. doi: 10.1002/jex2.70063 (PMC12269532; doi:10.1002/jex2.70063)
Supplement: Supplementary file 4 — Supplemental Figure S1. mRNA‐seq analysis of hypoxia hMSCs. Supplemental Figure S2. Original Western blot image for Figure 2D. Supplemental Figure S3. Examples of proteins differentially expressed in hMSCs and the secreted EVs: Cell/EV enriched proteins. Supplemental Figure S4. GO annotation of EV‐hypoxia only proteins (508) by proteomics. Supplemental Figure S5. Examples of proteins differentially expressed in hypoxia EVs and the normoxia EVs: EV (hypoxia/normoxia) enriched proteins. Supplemental Figure S6. Analysis of transmembrane protein before and after EV sonication. Supplemental Figure S7. Examples of proteins differentially expressed in hypoxia EVs before and after sonication: EV (before/after sonication) enriched proteins. Supplemental Figure S8. MRI to assess the distribution of labelled EVs and hMSCs at the ischemic lesion site. Supplemental Figure S9. 1H MRI analysis of hypoxia hMSC and EV treated rats following MCAO. Supplemental Figure S10. Pathway analysis of proteins related to HIF signaling. Supplemental Table S1. Donor information of bone marrow derived hMSCs. Supplemental Table S2. GO analysis (top) and KEGG analysis (bottom) for hypoxia versus normoxia hMSC proteomics datasets. Supplemental Table S3. The NTA result and protein quantification of each type of EVs. Supplemental Table S4. The miR and library quantification of each type of EVs. Supplemental Table S5. A list of mostly upregulated or downregulated miRNAs in the EVs. Supplemental Table S6. A list of proteins related to negative regulation of angiogenesis. [file JEX2-4-e70063-s001.docx]

**Supplemental Information**

**Cargo Analysis and MRI-based Therapeutic Assessment of Iron Oxide Labeled Extracellular Vesicles of Hypoxia Human Stem Cells in Ischemic Stroke**

Shannon Helsper^1,2,#^, Li Sun^1,3,#^, Richard Jeske^1^, Chang Liu^1^, Jacob Athey^1^, Xuegang Yuan^1,2^, Samuel C. Grant^1,2, *^, Yan Li^1,*^

^1^ Chemical & Biomedical Engineering, FAMU-FSU College of Engineering, Florida State University, Tallahassee, FL United States

^2^ National High Magnetic Field Laboratory, Florida State University, Tallahassee, FL United States

^3^ Department of Biomedical Sciences, Florida State University College of Medicine, Tallahassee, FL United States

^#^These two authors contributed equally to this work.

**Table of contents**

**Supplemental Figure S1.** mRNA-seq analysis of hypoxia hMSCs.

**Supplemental Figure S2**. Original Western blot image for Figure 2D.

**Supplemental Figure S3.** Examples of proteins differentially expressed in hMSCs and the secreted EVs: Cell/EV enriched proteins.

**Supplemental Figure S4.** GO annotation of EV-hypoxia only proteins (508) by proteomics.

**Supplemental Figure S5.** Examples of proteins differentially expressed in hypoxia EVs and the normoxia EVs: EV (Hypoxia/normoxia) enriched proteins.

**Supplemental Figure S6.** Analysis of transmembrane protein before and after EV sonication.

**Supplemental Figure S7.** Examples of proteins differentially expressed in hypoxia EVs before and after sonication: EV (before/after sonication) enriched proteins.

**Supplemental Figure S8.** MRI to assess the distribution of labeled EVs and hMSCs at the ischemic lesion site.

**Supplemental Figure S9.** ^1^H MRI analysis of hypoxia hMSC and EV treated rats following MCAO.

**Supplemental Figure S10.** Pathway analysis of proteins related to HIF signaling.

**Supplemental Table S1.** Donor information of bone marrow derived hMSCs.

**Supplemental Table S2.** GO analysis (top) and KEGG analysis (bottom) for hypoxia vs normoxia hMSC proteomics datasets.

**Supplemental Table S3.** The NTA result and protein quantification of each type of EVs.

**Supplemental Table S4.** The miR and library quantification of each type of EVs.

**Supplemental Table S5.** A list of mostly upregulated or downregulated miRNAs in the EVs.

**Supplemental Table S6.** A list of proteins related to negative regulation of angiogenesis.

**Supplemental Data files (excel) S1.** DEGs of hypoxia and normoxia hMSCs from mRNA-Seq.

**Supplemental Data files (excel) S2.** Proteomics data of hMSCs and the secreted EVs.

**Supplemental Data files (excel) S3.** Correlation of mRNA with the cell protein (RNA-Protein).

**Supplemental Figure S1. mRNA-seq analysis of hypoxia hMSCs.** (A) Heatmap of differentially expressed genes (DEGs); (B) Volcano plot of DEGs with blue representing downregulated DEGs by hypoxia and red representing upregulated DEGs by hypoxia.

**
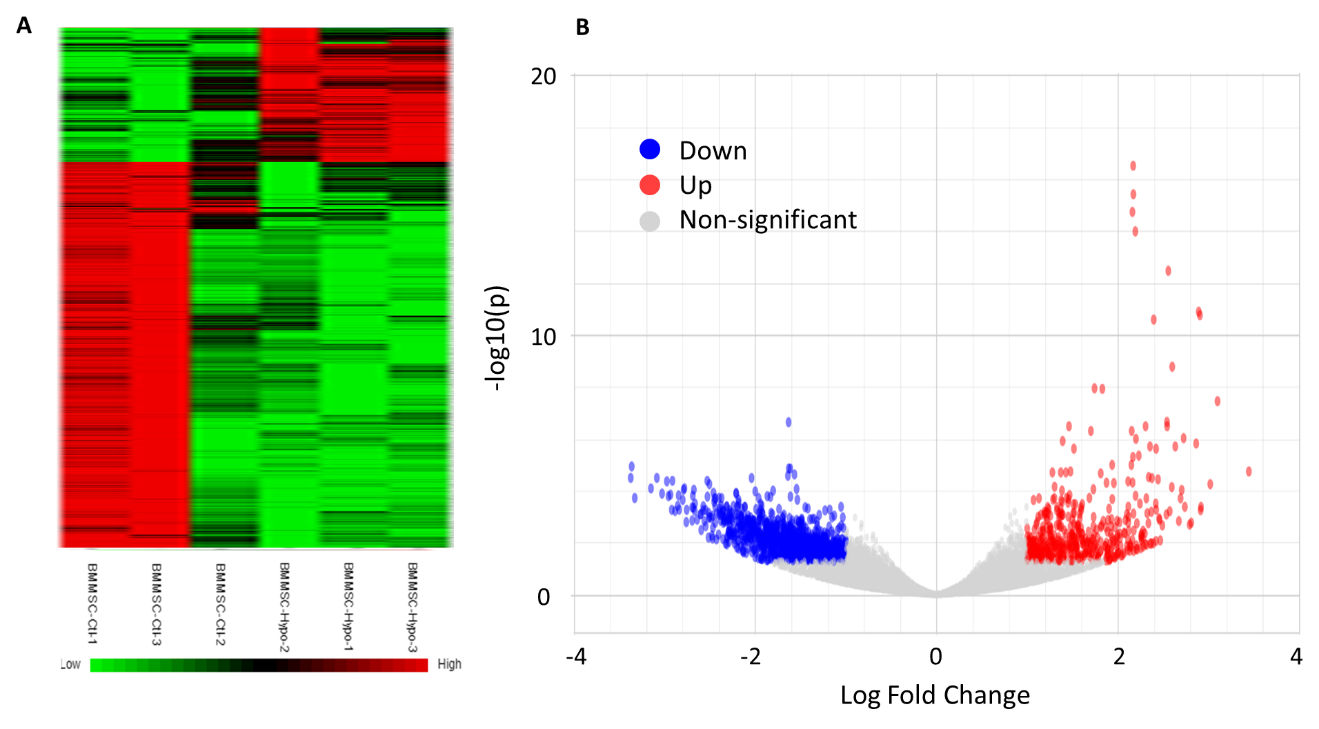
**

**Supplemental Figure S2. Original Western blot image for Figure 2D.**

**
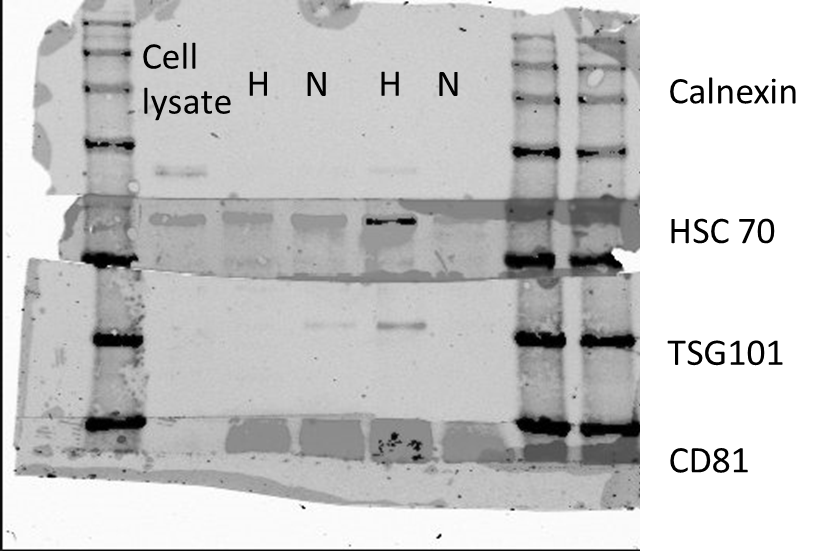
**

**Supplemental Figure S3. Examples of proteins differentially expressed in hMSCs and the secreted EVs: Cell/EV enriched proteins.** (A) Cluster of tubulin beta chain were similarly expressed in cells and EVs; (B) filamin C was high in cells but low in EVs; (C) fibronectin was low in cells and high in EVs.

**
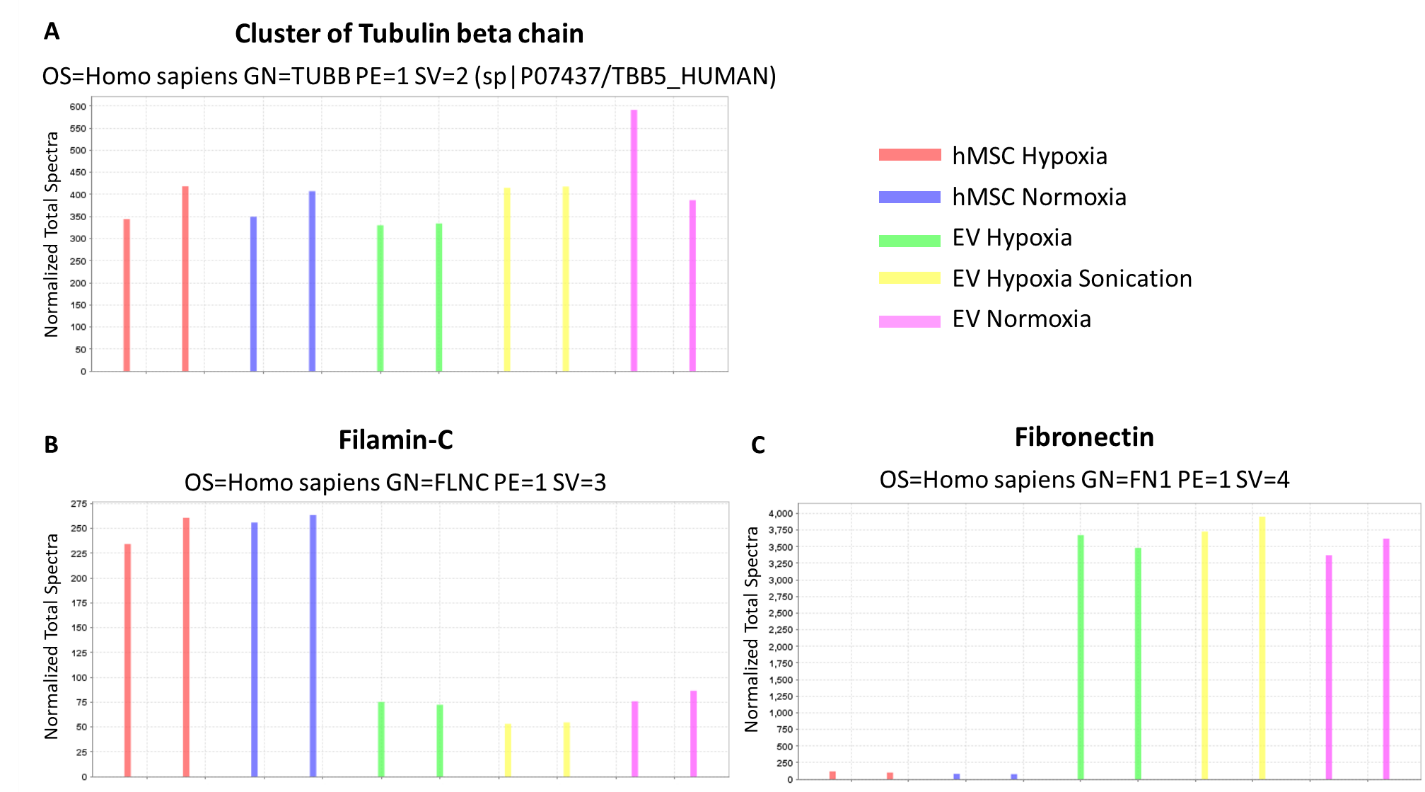
**

**Supplemental Figure S4. GO annotation of EV-hypoxia only proteins (508) by proteomics.**

**
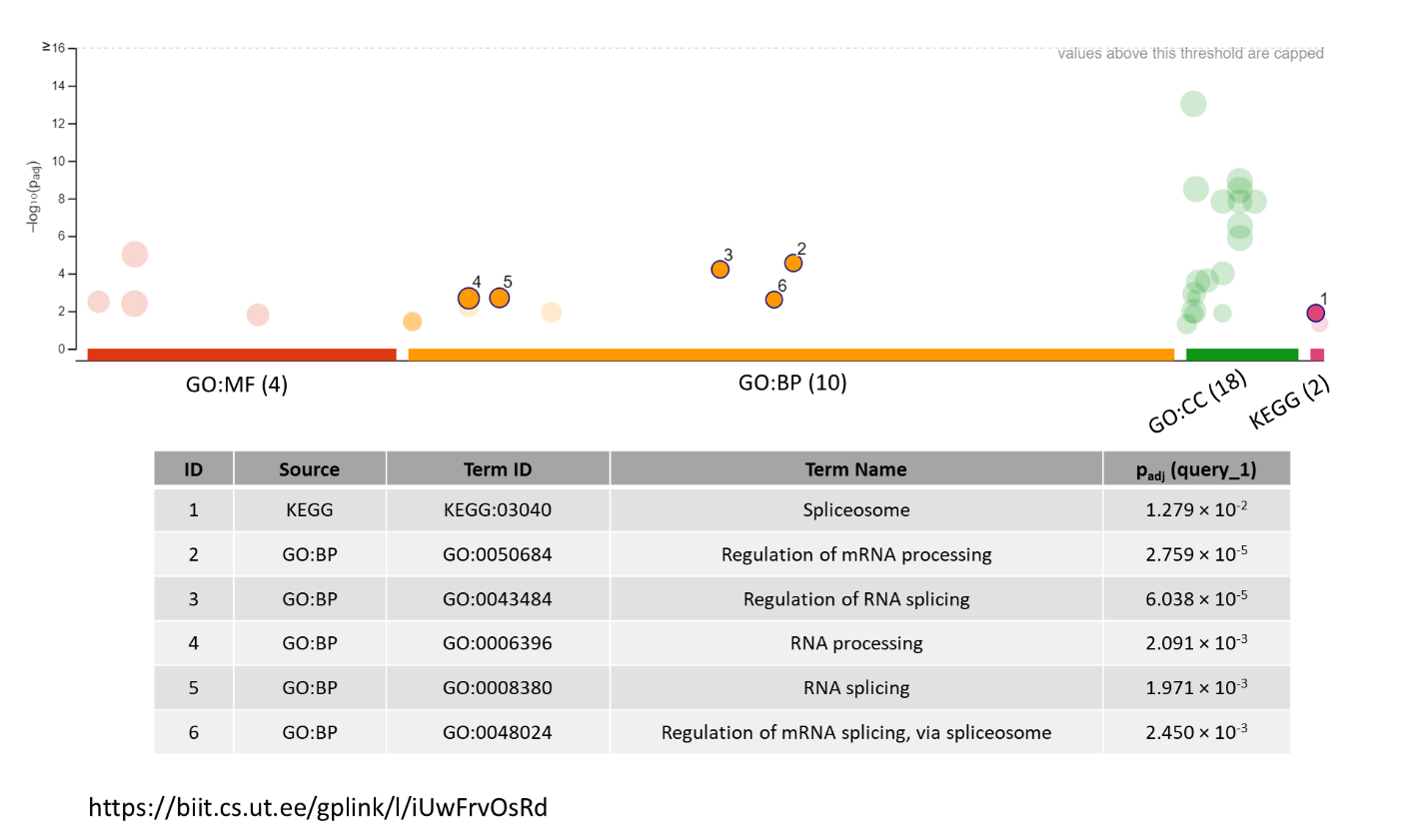
**

**Supplemental Figure S5. Examples of proteins differentially expressed in hypoxia EVs and the normoxia EVs: EV (Hypoxia/normoxia) enriched proteins.** (A) GAPDH was similarly expressed in both hypoxia and normoxia hMSCs as well as hypoxia and normoxia EVs; (B) Tenascin was high in normoxia EVs but low in hypoxia EVs; (C) glycogen phosphorylase was low in normoxia EVs and high in hypoxia EVs.

**
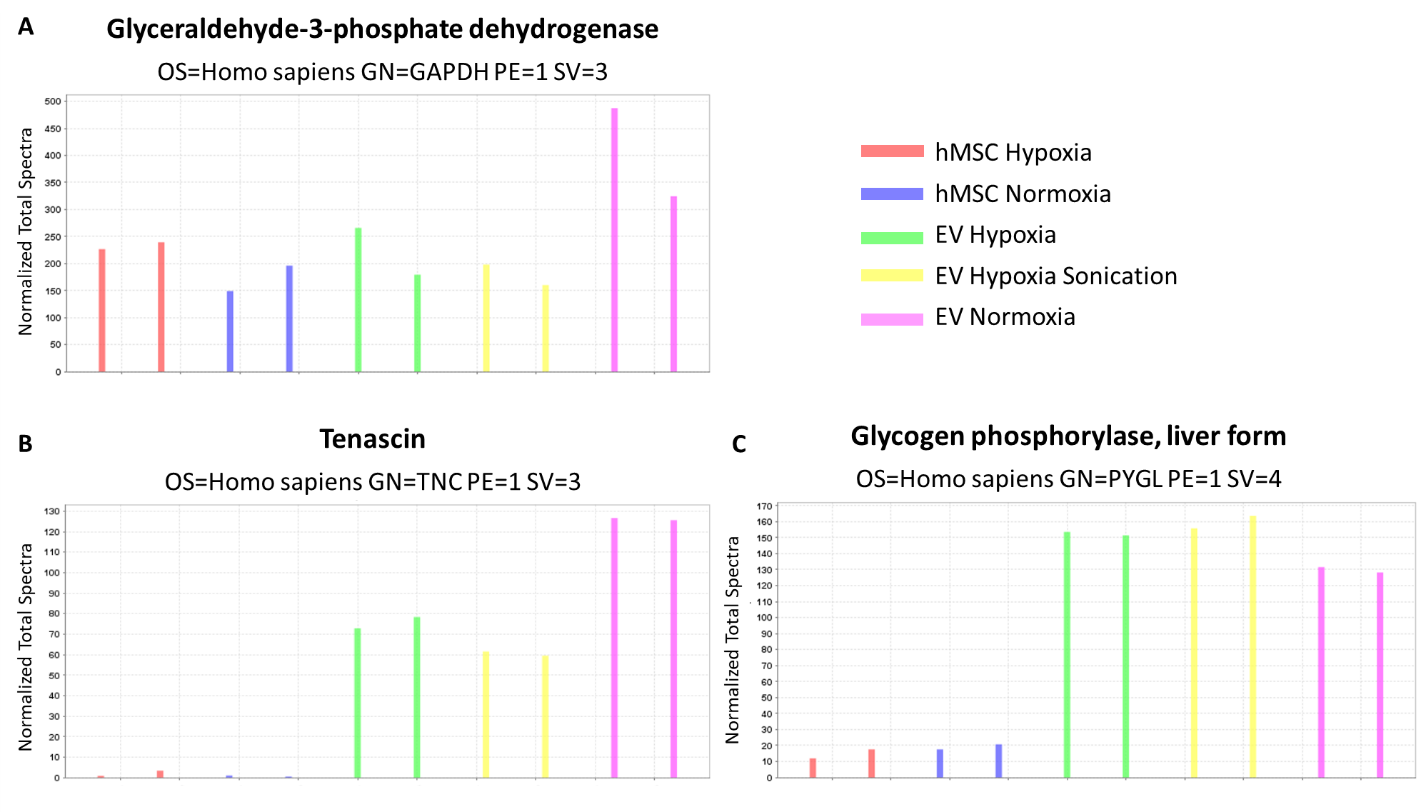
**

**Supplemental Figure S6. Analysis of transmembrane protein before and after EV sonication.** (A) Venn diagram of DEP for the three EV groups; (B) UniProtKB output; (C) ratio of transmembrane proteins to total protein number.

**
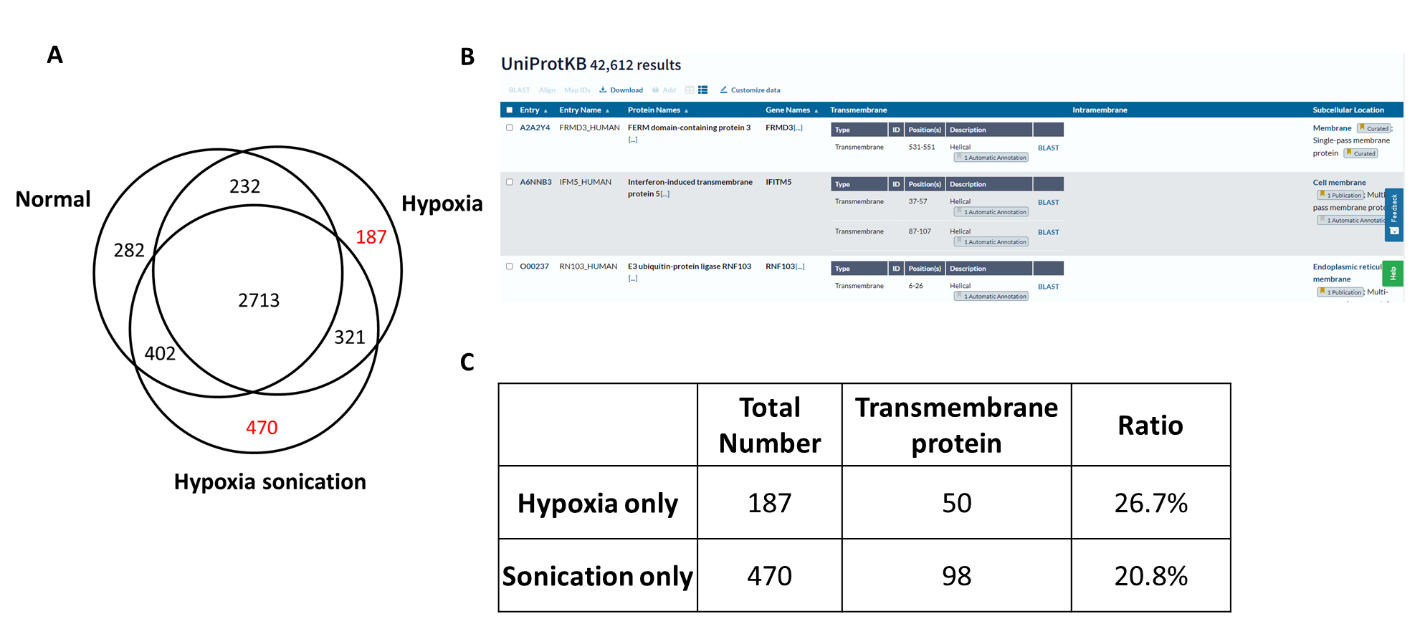
**

**Supplemental Figure S7. Examples of proteins differentially expressed in hypoxia EVs before and after sonication: EV (before/after sonication) enriched proteins.** (A) Fibulin-1, only present in EVs (not the cells) was downregulated after EV sonication; (B) Ninein, also only present in EVs (not the cells) was upregulated after EV sonication; (C) Integrin beta-1, present in both in cells and EVs, was upregulated after EV sonication.

**
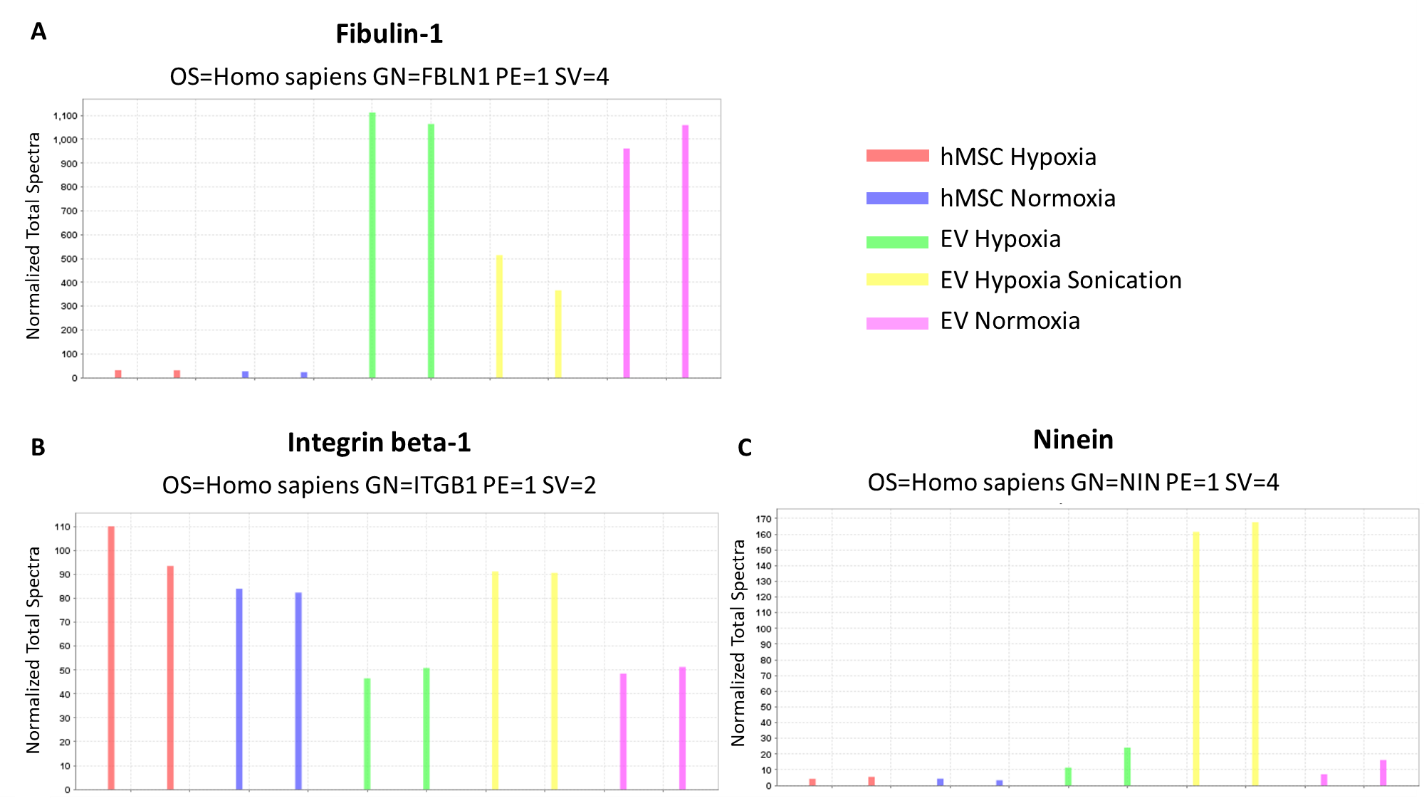
**

**Supplemental Figure S8. MRI to assess the distribution of labeled EVs and hMSCs at the ischemic lesion site.** ^1^H MRI of a rat injected with EVs (top) or hMSCs (bottom). EVs are seen in the ischemic hemisphere (blue circle) 3-h after injection, with full clearance seen 1-d post-injection. hMSCs are still prevalent in the ischemic hemisphere 1-day post injection, as indicated by the red circle.

**
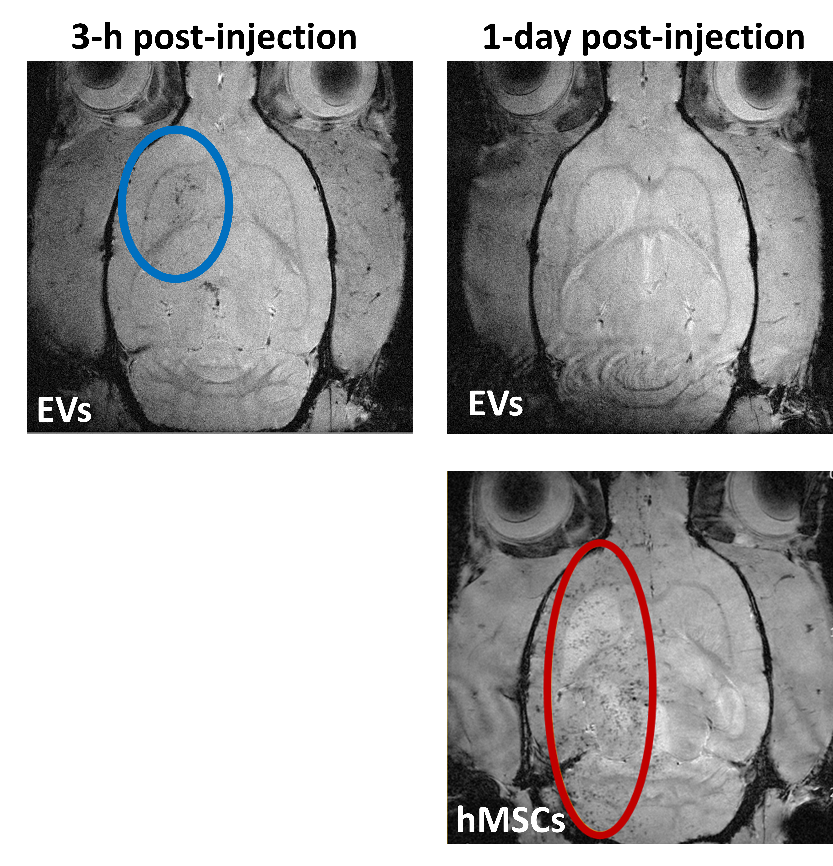
**

**Supplemental Figure S9. ^1^H MRI analysis of hypoxia hMSC and EV treated rats following MCAO.** Bar graphs representing (A) ischemic lesion volume and (B) fractional change in lesion volume over 21 days as defined by ^1^H MRI; (C) 3D rendering of representative EV administered rat on days 1 and (D) 21 with whole brain in yellow and ischemic lesion in orange.

**
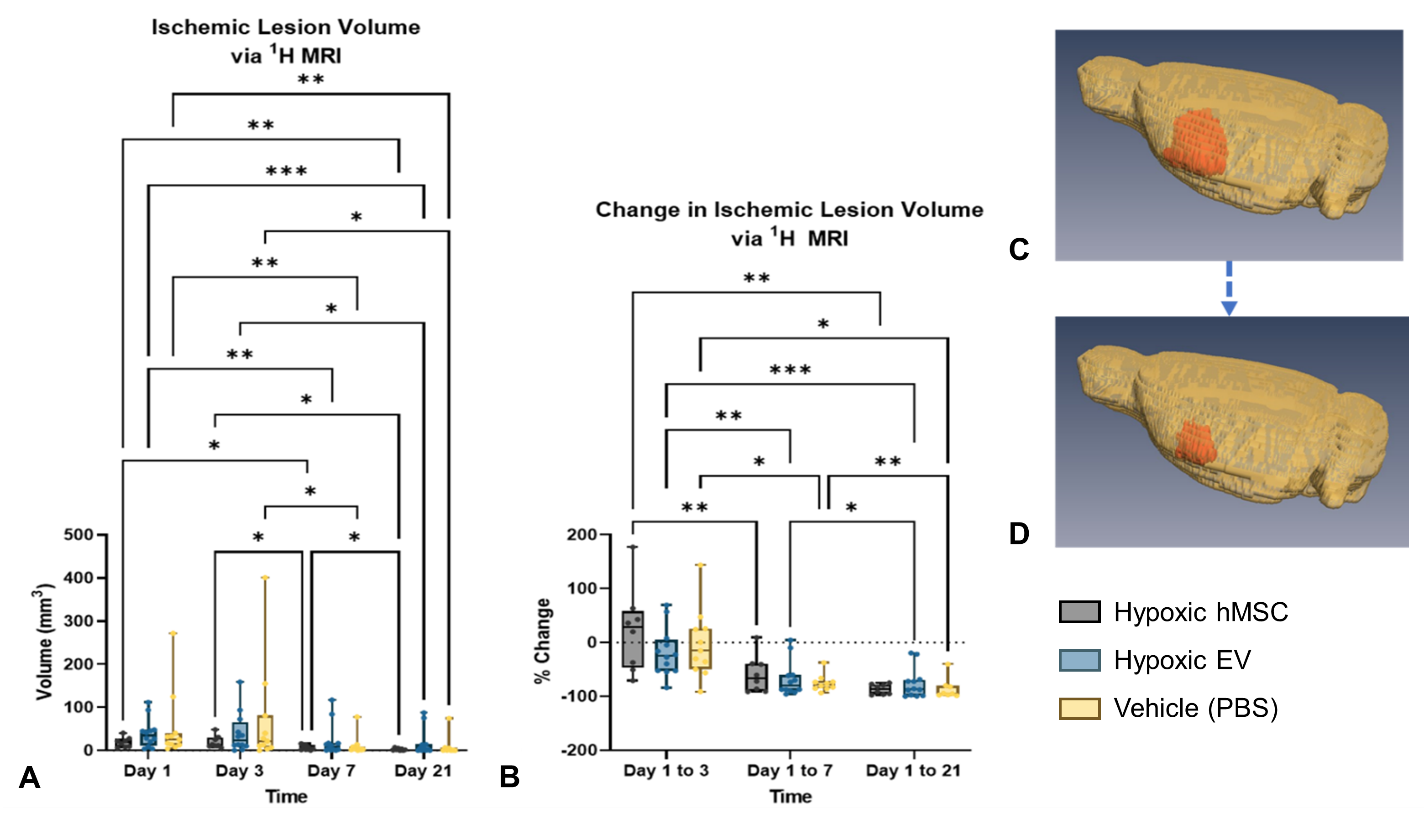
**

**Supplemental Figure S10. Pathway analysis of proteins related to HIF signaling.
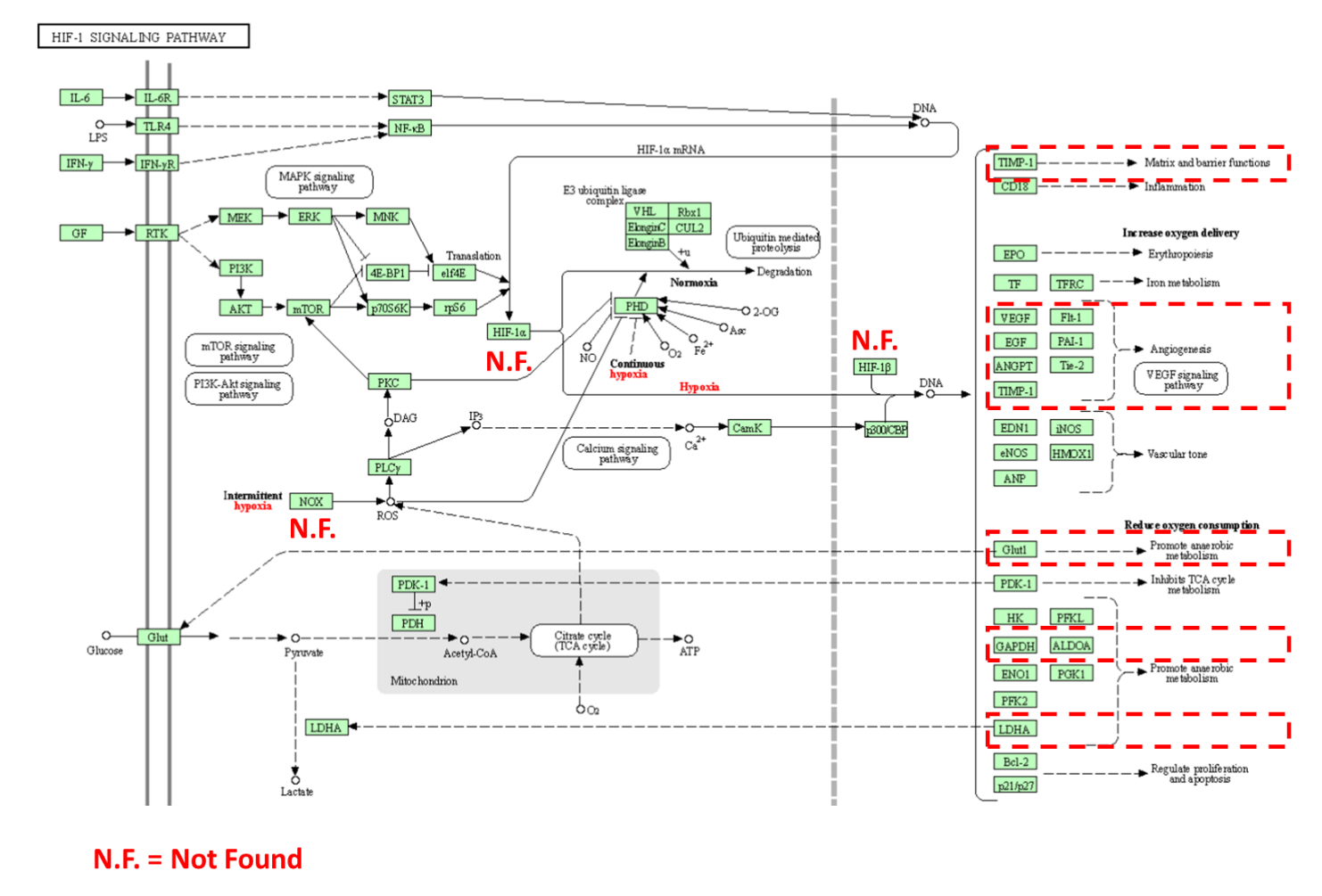
**

**Supplemental Table S1. Donor information of bone marrow derived hMSCs.**

| ID | Years | BMI | Race | Sex | note |
| --- | --- | --- | --- | --- | --- |
| 7051R | 33 y | NA | Caucasian | F | Tulane |
| 7052R | 20 y | NA | Caucasian | M | Tulane |
| 7038 | 30 y | NA | Caucasian | F | Tulane |

**Supplemental Table S2. GO analysis (top) and KEGG analysis (bottom) for hypoxia *vs* normoxia hMSC proteomics datasets.**

**
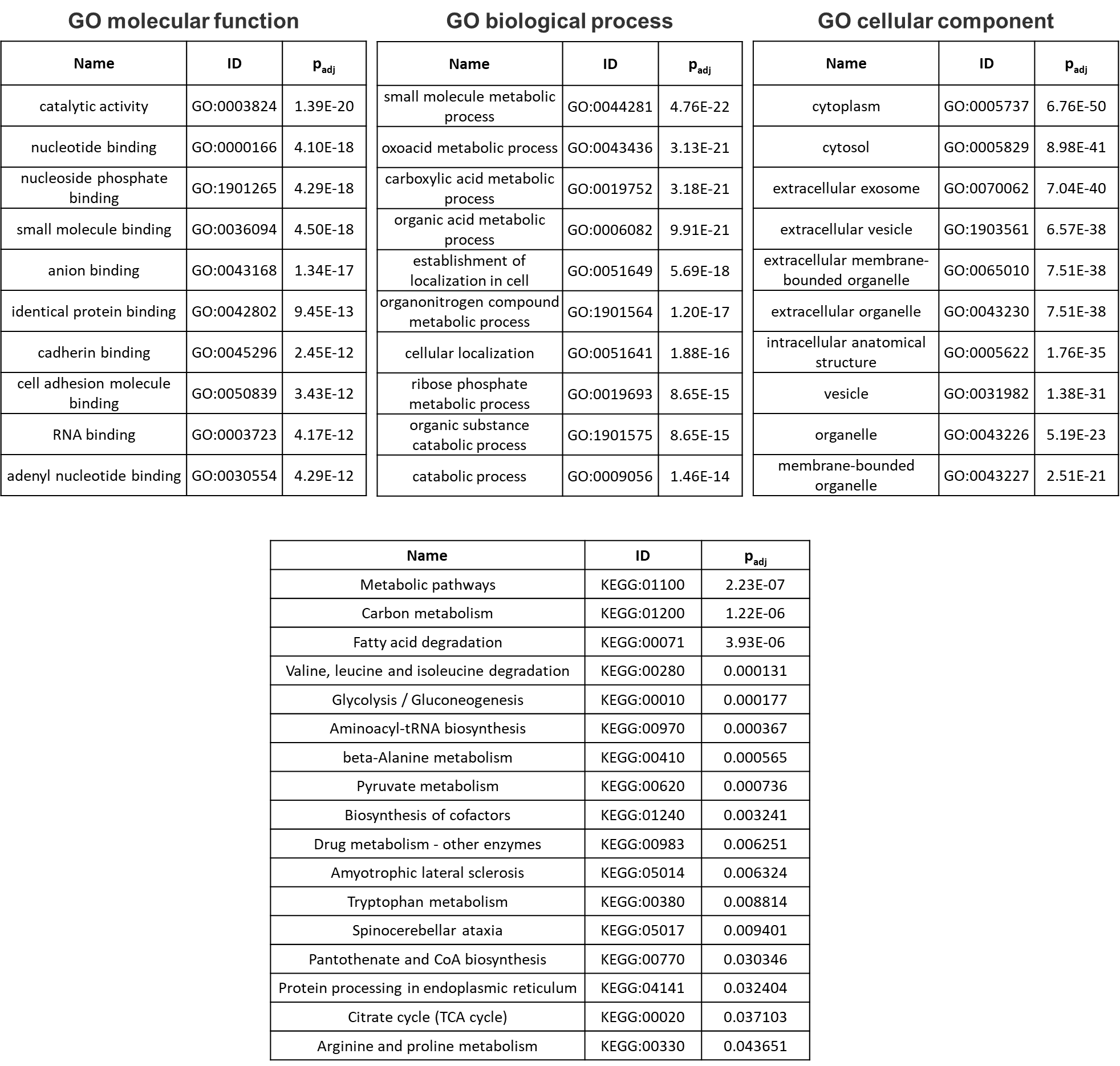
**

**Supplemental Table S3. The NTA result and protein quantification of each type of EVs.**

| Sample | Mean (nm) | Mode (nm) | Cal. Conc. (/µL) | Vol. (µL) | Protein Conc. (µg/µL) | Purity (/µg) | Total Protein (µg) | Protein per 10^10^ EVs | |
| --- | --- | --- | --- | --- | --- | --- | --- | --- | --- |
| MSC Ctl-1 | 137±0.8 | 106±5.1 | 1.6×10^10^ | 50 | 1.10 | 1.5×10^10^ | 54.8 | 0.685 µg | |
| MSC Ctl-2 | 128±3.3 | 112±1.5 | 2.6×10^10^ | 50 | 1.11 | 2.4×10^10^ | 55.5 | 0.427 µg | |
| MSC Hypo-1 | 143±1.8 | 124±3.4 | 3.5×10^10^ | 50 | 1.71 | 2.1×10^10^ | 85.3 | 0.485 µg | |
| MSC Hypo-2 | 152±7.2 | 128±5.4 | 3.2×10^10^ | 50 | 1.67 | 1.9×10^10^ | 83.3 | 0.521 µg | |
| Sample | **Mean (nm)** | **Mode (nm)** | **Cal. Conc. (/µL)** | **Vol. (µL)** | **Protein Conc. (µg/µL)** | **Purity (/µg)** | **Total Protein (µg)** | **Recovery by Protein** | **Recovery by EV counts** |
| Before Sonication | 221±6.1 | 176±3.6 | 1.1×10^10^ | 250 | 1.17 | 9.0×10^9^ | 291.9 | NA | NA |
| After Sonication | 161±3.9 | 144±3.9 | 4.1×10^9^ | 120 | 0.40 | 1.0×10^10^ | 48.4 | 24.2% | 27.4% |

**Supplemental Table S4. The miR and library quantification of each type of EVs.**

| Sample Name | Qubit miR conc. (ng/µL) | Library size (bp) | Library quantification by bioanalyzer (nM) |
| --- | --- | --- | --- |
| BMMSC-Nor-1 | - | 185 | 25.5 |
| BMMSC-Nor-2 | - | 182 | 7.6 |
| BMMSC-Nor-3 | - | 189 | 27.7 |
| BMMSC-Hypo-1 | 3.24 | 218 | 72.5 |
| BMMSC-Hypo-2 | 4.34 | 177 | 23.6 |
| BMMSC-Hypo-3 | - | 195 | 8.5 |

**Supplemental Table S5. A list of mostly upregulated or downregulated miRNAs in the EVs.**

| **miR Name** | **logFC** | **baseMean** | **adj.P.Val** |
| --- | --- | --- | --- |
| **hsa-miR-122-5p** | -4.54 | 5.4 | 0.02549 |
| **hsa-miR-3960** | -2.55 | 29.2 | 0.0086002 |
| **hsa-miR-133a-3p** | -2.54 | 256.3 | 1.45E-08 |
| **hsa-miR-93-5p** | -2.11 | 14.1 | 0.041544 |
| **hsa-miR-139-3p** | -2.01 | 28.8 | 0.0038973 |
| **hsa-miR-30d-5p** | -1.55 | 80.1 | 4.98E-05 |
| **hsa-miR-30e-5p** | -1.43 | 176.0 | 0.0038973 |
| **hsa-miR-6087** | -1.23 | 1386.4 | 0.010247 |
| **hsa-miR-181b-5p** | 1.25 | 37.0 | 0.046019 |
| **hsa-miR-224-5p** | 1.45 | 153.4 | 0.0038973 |
| **hsa-miR-7641** | 1.48 | 3839.9 | 0.0023533 |
| **hsa-miR-21-5p** | 1.89 | 602.8 | 3.32E-08 |
| **hsa-miR-146b-5p** | 1.89 | 21.8 | 0.0086002 |
| **hsa-miR-199a-3p** | 1.99 | 823.0 | 1.08E-06 |
| **hsa-miR-199b-3p** | 1.99 | 823.0 | 1.08E-06 |
| **hsa-miR-31-5p** | 2.09 | 12.6 | 0.033465 |
| **hsa-miR-664a-3p** | 3.48 | 12.6 | 0.0010676 |

**Supplemental Table S6. A list of proteins related to negative regulation of angiogenesis.**

**
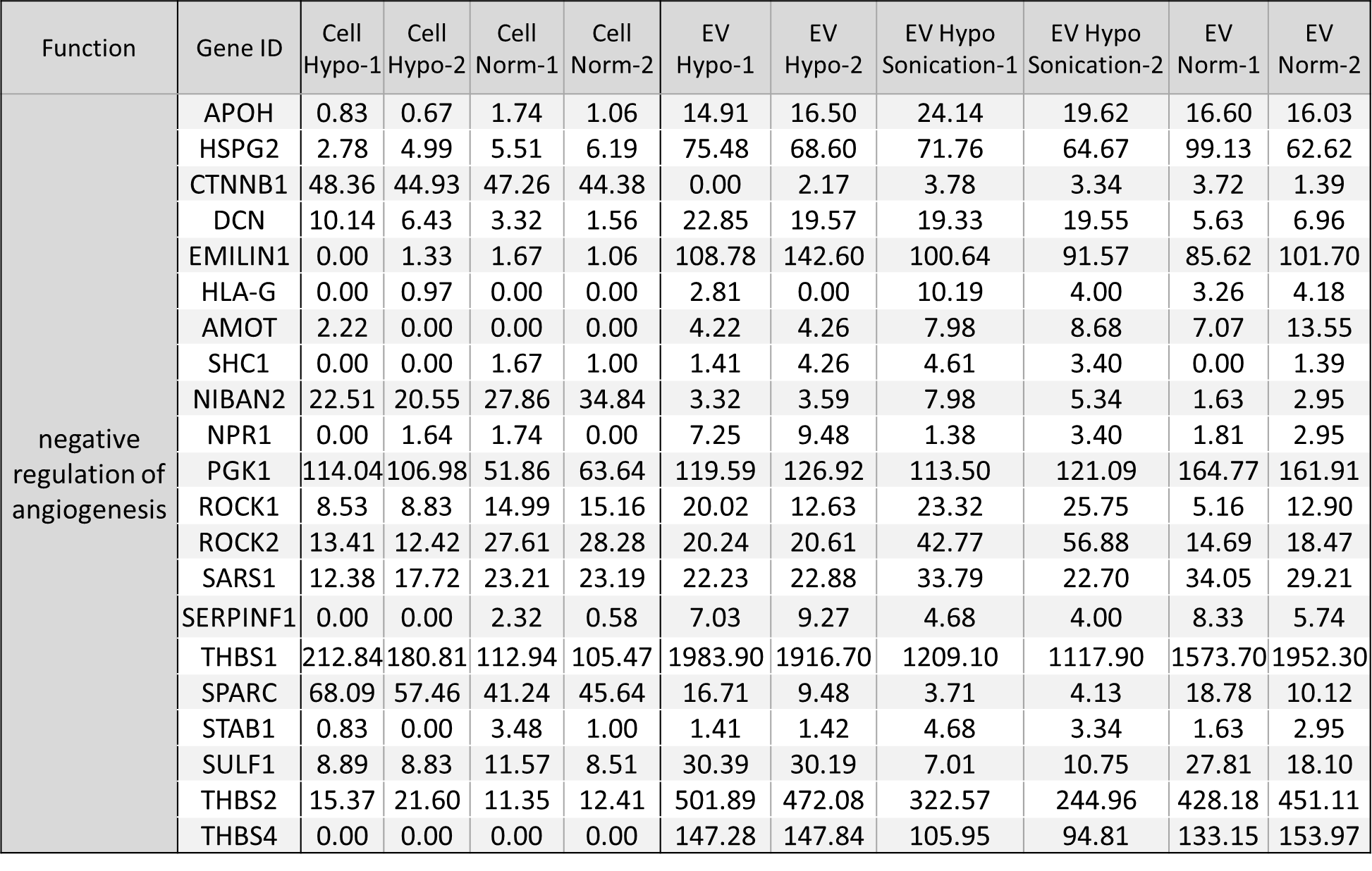
**

**Data files:**

**Supplemental excel spreadsheet S1.** DEGs of hypoxia and normoxia hMSCs from mRNA-Seq

**Supplemental excel spreadsheet S2.** Proteomics data of hMSCs and the secreted EVs

**Supplemental excel spreadsheet S3.** Correlation of mRNA with the cell protein (RNA-Protein)
